# Accessing Metal‐Containing Species in Tin–Lead Perovskite Precursor Solutions via Molecular Strategies Guided by the Hard–Soft Acid–Base Principle

**DOI:** 10.1002/anie.202514010

**Published:** 2025-08-19

**Authors:** Shuaifeng Hu, Xinru Sun, Wentao Liu, Luca Gregori, Pei Zhao, Jorge Pascual, André Dallmann, Akash Dasgupta, Fengjiu Yang, Guixiang Li, Mahmoud Aldamasy, Silver‐Hamill Turren‐Cruz, Marion A. Flatken, Sheng Fu, Yasuko Iwasaki, Richard Murdey, Armin Hoell, Susan Schorr, Steve Albrecht, Shangfeng Yang, Antonio Abate, Atsushi Wakamiya, Filippo De Angelis, Meng Li, Henry J. Snaith

**Affiliations:** ^1^ Clarendon Laboratory, Department of Physics University of Oxford Oxford OX1 3PU UK; ^2^ Key Laboratory for Special Functional Materials of Ministry of Education, School of Nanoscience and Materials Engineering Henan University Kaifeng 475004 P.R. China; ^3^ Helmholtz‐Zentrum Berlin für Materialien und Energie GmbH Hahn‐Meitner‐Platz 1 14109 Berlin Germany; ^4^ Department of Chemistry, Biology and Biotechnology University of Perugia Via Elce di Sotto 8 Perugia 06123 Italy; ^5^ Research Center for Computational Science Institute for Molecular Science Okazaki 444‐8585 Japan; ^6^ Polymat University of the Basque Country UPV/EHU Donostia‐San Sebastian 20018 Spain; ^7^ Institut für Chemie Humboldt‐Universität zu Berlin Berlin Germany; ^8^ School of Materials Science and Engineering Southeast University Nanjing Jiangsu 211189 P.R. China; ^9^ Instituto de Ciencia de los Materiales (ICMUV) Universitat de Valencia Paterna 46980 Spain; ^10^ School of Physics and Electronic Science, Engineering Research Center of Nanophotonics and Advanced Instrument Ministry of Education East China Normal University Shanghai 200062 P.R. China; ^11^ Institute for Chemical Research Kyoto University, Gokasho Uji Kyoto 611‐0011 Japan; ^12^ Key Laboratory of Materials for Energy Conversion, Anhui Laboratory of Advanced Photon Science and Technology, Department of Materials Science and Engineering University of Science and Technology of China Hefei Anhui 230026 P.R. China; ^13^ Department of Chemical, Materials and Production Engineering University of Naples Federico II Piazzale Tecchio 80 Fuorigrotta Naples 80125 Italy; ^14^ SKKU Institute of Energy Science and Technology (SIEST) Sungkyunkwan University Suwon 440‐746 South Korea

**Keywords:** Crystallisation, Lewis acid and base, Metal centre, Perovskite, Photovoltaics, Solution chemistry

## Abstract

The properties of metal‐centred species in metal halide perovskite precursor solutions substantially influence the formation and evolution of colloidal particles, which in turn dictate the crystallisation process and the film quality. In this work, we assess the “hard” and “soft” Lewis acid characteristics of Sn^2+^ and Pb^2+^ cations as a strategy to modulate the chemical environment of these metal‐containing species in mixed‐metal tin–lead perovskite precursor solutions. We observe enhanced simultaneous access to both metal centres upon adding compounds with functional groups suggested by the hard–soft acid–base principle. Theoretical calculations suggest that the hard base carboxyl group preferentially interacts with Sn^2+^‐based species, while the softer base thiol group also targets Pb^2+^‐based species. By effectively accessing and manipulating possible classes of inorganic species and their colloidal particle properties in the precursor solutions, we achieve 1.26 eV perovskite polycrystalline films exhibiting enhanced structural and optoelectronic quality, giving the best quasi‐Fermi level splitting values of up to 0.95 eV. As a result, the solar cell devices demonstrate efficiency values of up to 23.3% with an extended operational lifetime, retaining 80% of their initial efficiency after over 280 and 180 h of maximum power point tracking under simulated AM1.5G illumination at 25 and 65 °C, respectively.

## Graphical abstract

Molecular strategies guided by the hard–soft acid–base (HSAB) principle enable precise control over metal‐based species in perovskite precursor solutions. This approach enhances the quality of narrow‐bandgap (1.26 eV) mixed tin–lead perovskite films and improves the performance of the resulting solar cell devices.

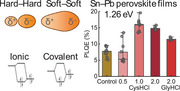

## Introduction

Metal‐containing species—studied across a wide spectrum of organic and inorganic chemistry^[^ ^1^, ^2^ ^]^—are also crucial components of metal halide perovskite precursors and their solutions, which are used in the deposition of thin films for fabricating optoelectronic devices that have garnered widespread interest.^[^ ^3^, ^4^, ^5^, ^6^, ^7^, ^8^ ^]^ In particular, mixed‐metal tin–lead (Sn–Pb) perovskites inherently exhibit greater complexity in B‐site‐related aspects compared to their neat lead‐ or tin‐based counterparts, suggesting considerable room for study.^[^ ^4^ ^]^ For optoelectronic device applications, the bandgap bowing effect (nonlinear bandgap change)^[^ ^9^, ^10^, ^11^ ^]^ in mixed‐metal Sn–Pb perovskite materials enables them to respond to electromagnetic radiation with energies as low as around 1.2 eV.^[^ ^9^, ^12^, ^13^, ^14^ ^]^ This narrow‐bandgap nature makes them highly suitable for fabricating efficient single‐ and multi‐junction solar cells^[^ ^4^, ^14^, ^15^, ^16^, ^17^, ^18^, ^19^, ^20^, ^21^ ^]^ and near‐infrared emitters/detectors, which are applicable in energy conversion, sensing and imaging technologies.^[^ ^22^, ^23^, ^24^, ^25^, ^26^, ^27^, ^28^ ^]^

The development of these materials and their devices has nevertheless been hindered by the limited understanding of their controllable thin film fabrication and quality retention, especially given the rapid crystallisation dynamics and the facile oxidation of Sn^2+^ to Sn^4+^.^[^ ^29^, ^30^, ^31^, ^32^, ^33^ ^]^ To deposit high‐quality thin films, the field has explored Lewis bases^[^ ^34^, ^35^ ^]^—a class of molecules that can establish interactions through electron/lone‐pair donating to the metal cations—to enable an intermediate stage for improved control of processing.^[^ ^34^ ^]^ Some examples are the introduction of dimethyl sulfoxide (DMSO) as a solvent for perovskite deposition,^[^ ^36^, ^37^, ^38^ ^]^ urea^[^ ^39^ ^]^ (or thiourea^[^ ^40^ ^]^), acetate^[^ ^41^ ^]^ and pyrrolidone^[^ ^42^ ^]^‐based molecules for solution^[^ ^40^, ^43^, ^44^ ^]^ and hybrid evaporation‐solution processes,^[^ ^45^ ^]^ or diamines^[^ ^46^ ^]^ and zwitterionic compounds^[^ ^47^ ^]^ to regulate the solution‐based crystal growth and defect passivation. Based on the existing literature,^[^ ^4^, ^16^, ^29^, ^30^, ^48^ ^]^ we note that in these mixed‐metal Sn–Pb perovskites, the strategies applied are largely focused on manipulating the Sn‐centred species,^[^ ^4^, ^21^, ^29^, ^41^, ^49^, ^50^, ^51^ ^]^ leaving the Pb^2+^‐based counterparts less explored. As learnt from the early time studies of the field,^[^ ^37^ ^]^ high‐quality neat Pb perovskite films are obtained through the introduction of intermediate phases composed of the solvent molecule(s) and metal‐centred species. In mixed metal systems, thus, understanding the individual roles of metal centres—potentially spanning multiple classes—and developing methods to tune the environment of each metal would unlock new possibilities. The simultaneous and controlled manipulation of the metal centres could avoid potential heterogeneities and compositional gradients, which are critical in systems with structural complexity.

**Figure 1:**
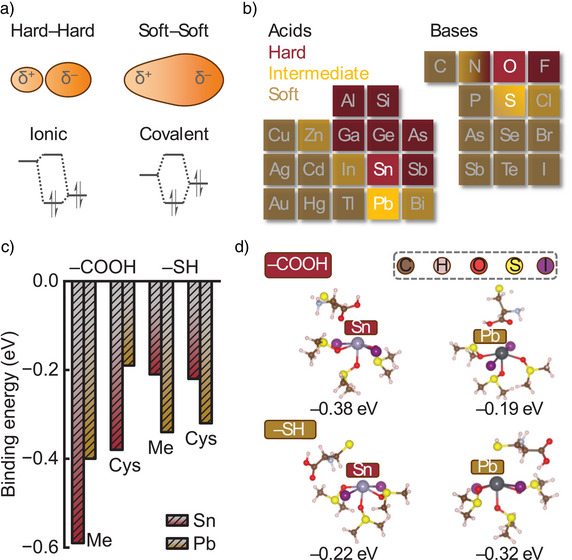
HSAB principle in theory: a) Bonding characteristics (top) and HOMO–LUMO interactions (bottom) for hard–hard and soft–soft cases. b) Hard–soft trends for acids and bases. c) DFT calculated binding energies between the CH_3_COOH/CH_3_SH (Me) (Cysteine, Cys) and SnI_2_•(DMSO)_3_/PbI_2_•(DMSO)_3_ complexes, with ─COOH or ─SH binding to the metal centres. d) DFT‐optimised configurations of cysteine and SnI_2_•(DMSO)_3_/PbI_2_•(DMSO)_3_ complexes with ─COOH or ─SH bound to the metal centres. The binding energies are provided below the optimised structural configurations.

In particular, Pb^2+^ and Sn^2+^ present intrinsic chemical differences in terms of their hard–soft characteristics as Lewis acids, thus making the hard–soft acid–base (HSAB) principle a tool with great potential to rationalise the behaviour of these metal cations. This empirical approximation describes acids and bases as hard for small, highly electronegative and non‐polarisable atoms/ions and as soft for big, low electronegative and polarisable ones with a more diffuse electron distribution (Figure 1a). Hard bases tend to bind hard acids, forming hard–hard species that establish affiliations through electrostatic interactions in a more ionic‐character bond, opposite to soft acids and bases that will tend to form soft–soft species with a higher covalent nature. This relative affinity is ultimately determined by the symmetry and energy of the frontier orbitals (HOMO (highest occupied molecular orbital) for the base and LUMO (lowest unoccupied molecular orbital) for the acid), which influence the strength of the resulting bond, as well as by the acidity/basicity level. Therefore, identifying the respective acids and bases in solutions as hard or soft will enable a prediction of their chemical affinities and the products being yielded. According to these characteristics, we can classify the different elements and their oxidation states as harder or softer species (Figure 1b). This also enables us to select ions or functional groups to design organic compounds for a preferential affinity towards a specific metal cation, according to its hard–soft nature.^[^ ^52^ ^]^ For our particular case, Sn^2+^ is a harder acid than the softer Pb^2+^, due to its higher charge density and lower polarisability despite their similar electronegativity values.

In previous studies, we observed the preference of the carboxyl group (─COOH) to coordinate with Sn^2+^ centres in mixed‐metal Sn–Pb perovskite systems, both in solution^[^ ^18^ ^]^ and at their surface sites of thin films.^[^ ^53^ ^]^ In addition, we also found that the Sn^4+^, oxidised from Sn^2+^, in solution can be effectively captured by, for instance, halide anions,^[^ ^54^ ^]^ based on the affinity variation raised by the charge‐induced Lewis acid hardness (a higher charge in the nucleus will make the cation harder). Thus, HSAB characteristics offer a simple and effective way to desirably access specific metal‐centred species and tune their chemical environment. In this work, we present a set of guidelines to access metal‐centred species in mixed‐metal Sn–Pb perovskite precursor solutions through the introduction of multifunctional molecules bearing basic groups that show a tunable preference towards Sn^2+^ and Pb^2+^. As suggested by density functional theory (DFT) calculations, the Sn^2+^ cation with a hard Lewis acid level will be preferentially bound with the electron/lone pair donating moieties that show hard Lewis basicity. Conversely, the Pb^2+^ cation tends to be more favourable towards softer Lewis bases. To verify this, we select different ammonium‐based salts and derivatives containing the hard ─COOH and/or soft (relatively) thiol (─SH) groups, which, as shown by solution characterisations, demonstrate an enhanced ability to concurrently manipulate the environment of both Sn^2+^ and Pb^2+^, owing to their respective chemical affinities. This control over metal‐containing species results in thin films with improved morphological and crystalline quality, enhanced optoelectronic properties and well‐performing photovoltaic devices.

## Results and Discussion

### Design Strategy

In our previous work, we comprehensively explored the precursor solution systems of the Sn–Pb perovskites modified with the amino acid, ammonium and carboxyl acid functionalities individually.^[^ ^18^ ^]^ Based on a series of characterisations, we revealed that the desirable effect of the modification from the ammonium and carboxyl groups requires the presence of these two functional moieties introduced via a single molecule. Intriguingly, we observed a significant chemical interaction between the carboxyl group and the Sn^2+^‐based species, while the Pb^2+^ counterparts remained less affected by this functional group. Considering the HSAB principle and the accessibility of organic compounds, we select cysteine hydrochloride (CysHCl), a proteinogenic amino acid‐based halide salt, to target species of both metals. In addition, this compound has also been employed successfully in Sn–Pb perovskite films for surface modification^[^ ^55^ ^]^ or the in situ generation of a redox dimer in precursor solutions.^[^ ^56^ ^]^ We propose here that each of the functional groups in CysHCl plays a specific role. Thanks to its enhanced polarity, the positively charged ammonium moiety is expected to improve optoelectronic film properties through effective defect passivation.^[^ ^18^ ^]^ Meanwhile, the carboxyl group preferentially interacts with Sn^2^⁺‐centred species, and the thiol moiety strengthens binding with Pb^2^⁺‐based species. This tailored molecular design enables precise control over solution properties, ultimately enhancing film quality.^[^ ^18^ ^]^

### Chemical Interactions

To prove the applicability of the hard–soft characteristics to this system, we perform calculations to evaluate the respective chemical affinity between functional groups. We first consider halogen‐metal complexes in DMSO, represented by solvated SnI_2_•(DMSO)_3_ and PbI_2_•(DMSO)_3_ complexes, which represent the possible stable species in solution suggested by the previous study.^[^ ^57^ ^]^ We investigate the interaction energy of these penta‐coordinated complexes with CH_3_COOH and CH_3_SH molecules, representing the individual binding groups of a cysteine molecule and, more generally, standing in as hard and soft Lewis bases, respectively (Figures 1c and S1). The carboxyl group interacts more favourably with the solvated Sn complex than with the Pb counterpart, with binding energies of −0.59 vs −0.40 eV. For the thiol group, in contrast, the trend is reversed, giving the binding energies of −0.21 and −0.34 eV for Sn‐ and Pb‐based species, respectively. In the case of cysteine, we also observe the same trend for the interactions of the optimised configurations (Figure 1c,d). This is further examined by two binding configurations of the perovskite lattices (Figure S2). Thus, these results suggest that, while both functional groups have favourable interactions with both metallic cations, the hard base ─COOH exhibits a certain preference for the hard acid Sn^2+^, while the soft base ─SH largely binds to the soft acid Pb^2+^, following the HSAB principle. In this way, cysteine can enhance the control over the environment of both Sn^2+^ and Pb^2+^ concurrently.

For experimental studies, we prepare mixed‐metal Sn–Pb precursor solutions, 1.8 M Cs_0.1_FA_0.6_MA_0.3_Sn_0.5_Pb_0.5_I_3_ (MA: methylammonium, FA: formamidinium), using DMSO/DMF (*N*,*N*‐dimethylformamide) mixture in a 1:3 volume ratio as solvent.^[^ ^49^ ^]^ During the preparation, we first observe that the addition of 1 mol% of CysHCl leads to a turbid perovskite precursor solution after about 1 h of stirring, and the turbidity increases with the amount of CysHCl added (Figure S3), suggesting the establishment of increased interactions between the amino acid and perovskite precursor materials, leading to precipitation/agglomeration. While the formation of a turbid solution may raise concerns about the stability of the precursor for thin‐film processing, we find that strictly limiting the dissolution time to approximately 30 min before deposition is critical. To understand which material(s) specifically lead to the phenomena, we prepare solutions with a series of material combinations of the precursor(s) and CysHCl. First, we mix CysHCl individually with each precursor material and observe that no combination leads to the appearance of a turbid solution (Figure S4). Thus, we aim for the opposite strategy, removing individual precursors from the full perovskite precursor solutions, while also considering the total removal of the organic (i.e., without both FAI and MAI) and metal cation parts (i.e., without both SnI_2_ and PbI_2_) (Figures S5 and S6). The photographs show that the solutions, which turned out to be noticeably not turbid, occur only in the cases without CsI, or SnI_2_ and PbI_2_, or SnF_2_. The level of turbulence also shows a concentration dependence, appearing to be clear when the full 1.8 M perovskite precursor solution was diluted down to about 0.8 M (Figure S7). To explore whether the observed turbidity could be attributed to the potential formation of low‐solubility CsF in solutions with high concentrations of inorganic components, we conduct further investigations into the solution behaviour. We prepare Sn–Pb perovskite precursor solutions without additives under study (control) and with the addition of GlyHCl and CysHCl—where CysHCl differs from GlyHCl by the presence of a ─CH_2_─SH group. All filtered solutions are observed at 0, 30, 60, 120 and 240 min to monitor changes in appearance (Figure S8). Initially (0 min), all solutions are clear. The control and GlyHCl‐containing solutions remain clear for the entire 240‐min period. In contrast, the CysHCl‐containing solution becomes noticeably turbid after 60 min and remains turbid up to 240 min. This suggests that the turbidity primarily arises from the ─CH_2_─SH group present in CysHCl. Our findings support the hypothesis that the introduction of a softer functional group enhances interactions between the amino acid salts and the inorganic precursor‐containing species in solution, where a small addition (5 mol%) of SnF_2_ appears to play a critical role.

**Figure 2:**
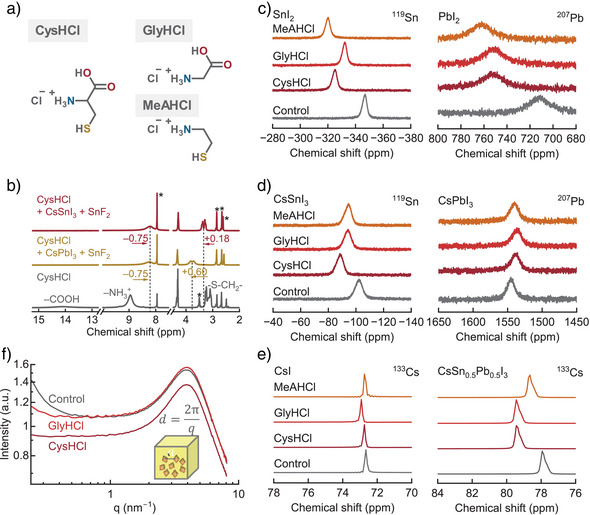
Solution chemistry characterisations. a) Chemical structures of cysteine hydrochloride (CysHCl), glycine hydrochloride (GlyHCl, without ─CH_2_─SH vs CysHCl) and 2‐mercapto­ethyl­amine hydrochloride (MeAHCl, without ─COOH vs CysHCl). b) ^1^H NMR of CysHCl and with the addition of CsPbI_3_ and CsSnI_3_ (asterisk is the signal from DMF). ^119^Sn and ^207^Pb NMR of c) MI_2_ and d) CsMI_3_ (M = Sn and Pb) without and with different additives. e) ^133^Cs NMR of CsI and CsSn_0.5_Pb_0.5_I_3_ without and with different additives. f) SAXS scattering curves of the mixed Sn–Pb perovskite precursor solutions prepared with no additive under study (control), individually with the addition of 1 mol% GlyHCl or CysHCl.

We then focus on understanding the interactions between the inorganic materials and the additives in the solution. We prepare and characterise the inorganic material solutions bearing CysHCl and its different derivatives using ^1^H NMR (Figure 2b). We focus on the inorganic materials due to the affinity of Cys to them based on their chemical nature and the above observations. The removal of the relatively large number of organic signals from hybrid perovskite compositions could reduce the possibility of having the proton signals of the additives overlap with the organic precursors. We mix CysHCl with the two different inorganic perovskite compositions (CsSnI_3_ and CsPbI_3_, with 5 mol% SnF_2_), analysing the change in each functional group when in the presence of Sn or Pb precursors. Due to the presence of Sn^2+^ from SnF_2_ in both samples, the proton of the carboxyl group at 14.1 ppm disappears after perovskite addition, suggesting strong coordination with Sn^2+^ cation.^[^ ^18^, ^53^ ^]^ The thiol proton does not show up in any spectra, which is expected to appear around 2–2.5 ppm, presumably due to a fast exchange rate with the small water content present in the solution.^[^ ^58^ ^]^ However, the protons of the carbon attached to the sulphur atom, which are differentiated due to their diastereotopic nature, are strongly shifted to the lower field. This deshielding of the protons necessarily comes from the increase of the electron‐withdrawing character of the sulphur atom, suggesting the electron deficiency in sulphur. This is most certainly due to its coordination with the metal cations in solution through electron sharing. Furthermore, the shift is much stronger for the Pb‐based perovskite (+0.60 ppm) than for the Sn‐based one (+0.18 ppm), suggesting stronger donating and affinity of the soft thiol group to softer Pb^2+^ in comparison to harder Sn^2+^. The ammonium protons (integrating for ∼3 units) also shift, but to more shielded positions, to 0.75 ppm lower in both cases. This implicates the interaction of the ammonium group with the perovskite precursors through the halide(s), which increases the electronic density around the ammonium protons.^[^ ^18^ ^]^ This interaction could also facilitate the arrangement of the amino acid cations on the surfaces of the perovskite particles. Thus, Cys cation presents evidently enhanced interactions with the perovskite precursors in solution through the three functional groups studied, i.e., ─NH_3_^+^, ─COOH, and ─SH, consistent with the theoretical modelling results.

To evaluate the existence of chemical preferences, according to the HSAB principle, for the carboxyl and thiol groups for Sn^2+^ and Pb^2+^ centres, respectively, we conduct liquid ^119^Sn and ^207^Pb NMR measurements. For a tight comparison, we prepare samples of 0.5 M PbI_2_, CsPbI_3_, SnI_2_ and CsSnI_3_ individually with the addition of 5 mol% of CysHCl, GlyHCl (without ─CH_2_─SH vs CysHCl) and MeAHCl (without ─COOH vs CysHCl) (Figure 2a). The ^207^Pb and ^119^Sn NMR results show a deshielding of the signal for every additive in the order of MeAHCl > CysHCl > GlyHCl for both PbI_2_ and SnI_2_ solutions (51, 41 and 40 ppm, and 27, 22 and 15 ppm, respectively) (Figure 2c,d). The downfield shift is caused by a partial replacement of the most electron‐donating ligand, DMSO, by a less‐donating one(s),^[^ ^18^ ^]^ which in this case would be any of the functional groups in the additives under study. This observation points out a stronger influence on the environment of both metallic cations by the additives containing the thiol group. In the case of CsSnI_3_ solutions, the order in downfield shift is altered to CysHCl > GlyHCl ≈ MeAHCl with smaller absolute changes (14, 8 and 8 ppm, respectively). These results suggest that the multifunctional cysteine moiety establishes overall stronger interactions with the perovskite precursors in solution. The stronger shift caused by the thiol group in the Sn spectra does not necessarily mean a more favourable binding that opposes the HSAB principle. Instead, it is a consequence of the S atom in the thiol group having a more available lone pair than the O in the carboxyl, which would increase its electron‐donating ability and thus increase the electron density on Sn^2+^ more effectively. Thus, the coordination of Sn^2+^ by the thiol can be energetically less favourable than by the carboxyl and still have a higher influence on its chemical shift. Nevertheless, these results unequivocally prove that both functional groups have the ability to coordinate Sn^2+^. Meanwhile, the differences are very subtle in the case of CsPbI_3_ solutions, with comparable chemical shift values for all the samples in a range of less than 10 ppm, relative to the large chemical shift range, e.g., 1545.16 ppm for the control. Interpreting the CsPbI_3_ solutions with a complete Pb‐based inorganic perovskite composition is more challenging, as the addition of the A‐site causes a substantial downfield shift (+830 ppm) due to the higher Pb–I coordination level.^[^ ^59^ ^]^ In this scenario, there is a reduced contrast in chemical shift effects between iodide ions and the functional groups of the additive, masking the specific effects of the additives for this particular case. Interestingly, we observe that in these solutions containing neat inorganic perovskite precursors, the thiol group also noticeably affects the chemical shift of Sn^2+^, in the same way as the carboxyl group affects the chemical shift of Pb^2+^. Thus, the proton and metal NMR results point out that the different functional groups exhibit soft–soft and hard–hard preferences towards the metal centres, though without strict selectivity. Both carboxyl and thiol groups form relevant interactions with Sn^2+^ and Pb^2+^ alike. These findings align with the DFT results in Figure 1c, which are compatible with HSAB‐based chemical preferences, while also supporting the occurrence of both hard–hard/soft–soft and hard–soft bindings, consistent with the negative energy values from the modelling in all cases. Overall, combining functional groups of designated nature within a single structure, such as in the cysteine moiety, enhances simultaneous access to the metal cations, enabling strengthened control over their chemical environment.

The solutions also contain the inorganic Cs^+^ cation, to which the studied additives could have a certain or high affinity. We evaluate any potential influence from it on the behaviour observed for the additives by measuring ^133^Cs NMR of CsI and CsSn_0.5_Pb_0.5_I_3_ solutions (Figure 2e). CsI solutions in the presence of the additives show negligible shifts (less than 0.2 ppm), indicating the lack of relevant coordination of the cation by the thiol and carboxyl groups due to the much larger size of this cation compared to Sn^2+^ and Pb^2+^, which have a similar ionic radius and thus allow their comparison in the present study. However, mixed‐metal‐containing CsSn_0.5_Pb_0.5_I_3_ solutions exhibit substantial shifts of Cs: 0.8 ppm for MeAHCl and 1.6 ppm for CysHCl and GlyHCl, suggesting that the influence of the additive on the inorganic perovskite colloidal particles is likely promoted through the B‐site metal‐centred species. This aligns with the critical role of Cs in perovskite colloidal formation, thus also clarifying the eventual turbid solution of the inorganic perovskites as observed.

Taking into account the notable preferential interactions of CysHCl with the mixed Sn–Pb perovskite precursors, we anticipate the ability of this additive to influence the colloidal properties of the mixtures and thus their crystallisation into thin films.^[^ ^18^ ^]^ Hence, we perform small‐angle X‐ray scattering (SAXS) to reveal the effect of CysHCl on the mixed Sn–Pb perovskite precursors in solution.^[^ ^60^, ^61^ ^]^ In Figure 2f, we compare the SAXS curves of 1 mol% CysHCl with the same amount of GlyHCl and a reference sample with no additive under study. We observe no difference in the position of the structure factor maximum, meaning that the recurring interparticle distance d among the three samples remains unchanged, located for all of them at a *q* of roughly 4 nm^−1^. However, at low *q* values, the introduction of additives leads to a flattening of the slope compared to the control, more pronouncedly for CysHCl. The negative slope at low *q*‐values can be assigned to the existence of larger structures, being uncovered in the scanned *q*‐range, with a broad size distribution. In contrast, the absence of this slope in combination with the presence of small particles/subunits indicates an arrangement of significantly larger scale. Therefore, these two additives seemingly share the potential to homogenise the size distribution of larger species (likely dominated by the inorganic precursors), suggesting a universal role of this class of additives, amino acid salts, for manipulating perovskite colloidal properties effectively.^[^ ^18^, ^20^ ^]^ This trend is evident in the concentration study for both additives, from 1 to 5 mol% (Figure S9). Meanwhile, for CysHCl, we obtain comparable results for any concentration, meaning that the positive effect on the colloids is obtained already at low concentrations compared to the gradual evolution of the GlyHCl case, suggesting that the thiol group is further promoting a homogeneous distribution of particles in solution. These results prove that we can ultimately influence the perovskite precursor colloidal properties by selecting the chemical nature of additive functionalisation for specific interactions in solution. In this case, further introducing a softer functional group with a high affinity to softer Pb^2+^ opens the door to modulating the environment of all classes of metal cation‐based species in solution more strongly, yielding a homogeneous distribution of particles in solution. This is expected to contribute to a homogenised nucleation and crystallisation for better thin film growth.

### Film Properties

**Figure 3:**
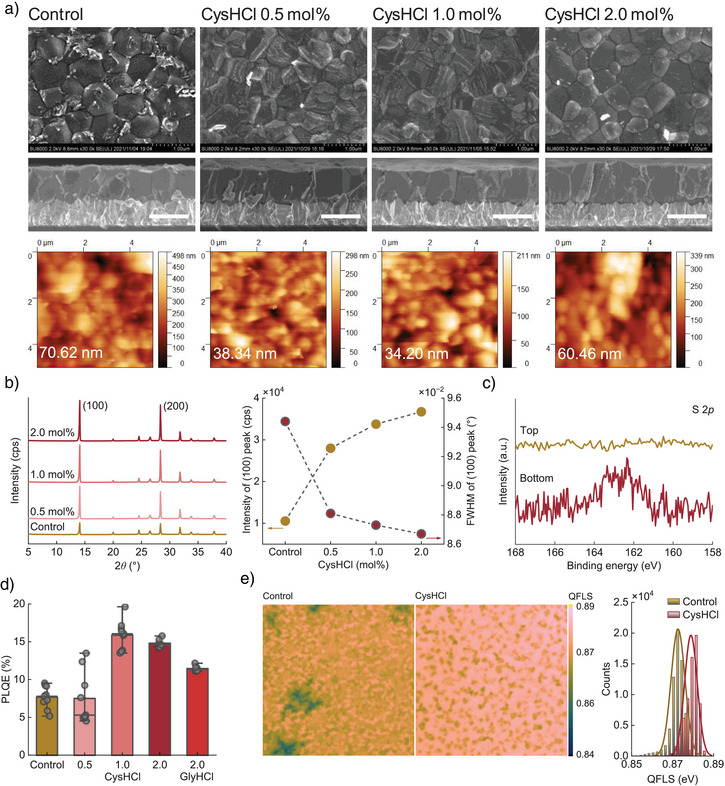
Film properties: a) Top‐view (top) and cross‐view (middle, scale bar: 1 µm) SEM images and topographic AFM (atomic force microscope) images (bottom, root mean square (RMS) roughness is provided), b) XRD patterns and the intensity and FWHM (full width of the half maximum) values of the (100) peaks of the perovskite films prepared with no particular additive (control) and with the addition of 0.5, 1.0 and 2.0 mol% CysHCl. c) XPS spectra of the S 2*p* core levels of the perovskite films prepared with the addition of 1 mol% CysHCl, with the samples probed from the top and bottom (using a film peeled off from the glass substrate) surfaces. d) PLQE of different perovskite films illuminated from the glass side with a 532 nm laser at a 1‐sun equivalent intensity for a 1.26 eV bandgap. Post‐treatment is applied to all the films. e) QFLS mapping (left) calculated from 1.2 mm × 1.2 mm PLQE images for the perovskite films recorded at open‐circuit conditions under illumination (equivalent to 1‐sun intensity for a 1.26‐eV bandgap) from the glass side, and QFLS distribution histogram (right) generated with values collected from the left‐side images.

To assess the impact of CysHCl on the morphology and crystallinity of perovskite films, we deposit thin films with varying additive concentrations and conduct scanning electron microscopy (SEM) and X‐ray crystallography diffraction (XRD) characterisations. The top‐view SEM of the control samples shows a compact thin film with a homogeneous distribution of grains (Figure 3a). It presents, however, bright deposits mainly in the region close to the grain boundaries, probably related to SnF_2_ residuals,^[^ ^62^ ^]^ which are less noticeable for CysHCl‐containing films. Compared to the rest of the films, the 2 mol% CysHCl films show more well‐defined boundaries of grains and grains with a cleaner surface and slightly larger size. The statistical grain size analysis also suggests a slight increase in the mean values with the increase of added CysHCl (Figure S10). The cross‐sectional SEM images show no significant difference among the examined films, giving a thickness of around 850 nm. We in addition interpret the RMS roughness from the topographic AFM images, yielding the values of 70.62, 38.34, 34.20, and 60.46 nm for the control, 0.5, 1.0, and 2.0 mol% CysHCl‐added films, respectively. For the CysHCl films, the increased roughness in the 2 mol% case is associated with the increased grain size and well‐defined grain boundaries, as suggested by the SEM image. Therefore, the introduction of CysHCl, particularly in moderate concentrations, leads to improvements in the perovskite thin film morphology.

The XRD patterns are dominated by two peaks of the films with the 2*ϑ* values centred at 14.2° and 28.4°, corresponding to the reflection of the (100) and (200) crystalline planes, respectively^[^ ^20^ ^]^ (Figure 3b). Upon the addition of the CysHCl, we observe a systematic intensity enhancement of the (100) peaks, with the 2 mol% CysHCl films showing a nearly fourfold intensity increase compared to the control, while the minor reflection at 2*ϑ* of 20.1° from (110) remained unchanged. In addition, the FWHM values of the (100) peaks decrease gradually with the increased addition of CysHCl, consistent with the accordingly increased grain size collected from the SEM images. We observe no considerable variations in the *d*‐spacing values of the lattice, no diffraction peaks detected at low 2*ϑ* values (5°–12°, a region where low‐dimensional species generally appear), and no feature changes in the absorption spectra of the films (Figure S11). These results suggest that introducing a reasonable amount of CysHCl through the perovskite precursor solution will not impact the integrity of the 3D crystalline structure of the as‐deposited films, while the molecules are thus more possibly attached to the crystalline surface at the grain boundary and interface regions. Overall, introducing CysHCl into the precursor solutions for depositing mixed‐metal Sn–Pb perovskite films enables substantial improvements in their morphology and crystallinity. These likely benefit from homogeneous nucleation and material propagation, thanks to the desirable chemical interactions and colloidal properties of the perovskite solutions induced by the CysHCl additive.

Driven by an interest in locating the additive in the films, we prepare the CysHCl films and probe from the exposed top surface and the buried bottom surface (of a film peeled off from the glass substrate^[^ ^63^ ^]^) using X‐ray photoelectron spectroscopy (XPS) measurements (Figure 3c). Based on the spectra of the S 2*p* core levels, we observe only noise‐level signals for the measurement of the top surface of the film. However, a definable peak appears for the films with the exposed bottom surface. This suggests that the Cys cations introduced into the perovskite precursor solution predominantly sediment at the buried region of the deposited films, following the observation and mechanism of our previous studies.^[^ ^18^, ^20^, ^64^ ^]^

We conduct macroscopic photoluminescence quantum efficiency (PLQE) measurements under the 1‐sun equivalent intensity of 1.26 eV bandgap (Figure 3d). We probe at least six different points from no less than two films of each condition. The statistical analysis of the results suggests that the films prepared with 1 mol% CysHCl show a mean PLQE value of 15.9%, substantially higher than that of the control and 0.5 mol% CysHCl films at 7.7% and 7.5%, respectively. The highest PLQE value obtained for the 1 mol% CysHCl films is up to 19.6%, yielding a quasi‐Fermi level splitting (QFLS) value (*qV*_OC_) of 0.95 eV, which corresponds to 96% of the radiative limit, 0.99 eV, for the 1.26 eV bandgap material.^[^ ^65^ ^]^ To the best of our knowledge, these are the best values reported for mixed Sn–Pb perovskite materials. With the 2 mol% addition of the additives, we observe slightly higher PLQE from CysHCl films compared to GlyHCl films, with mean values of 14.8% and 11.5%, respectively. We further map out the QFLS by calculating the imaged absolute PLQE of the control and 1 mol% CysHCl film using the setup we reported previously^[^ ^66^ ^]^ (Figure 3e). Compared to the control, the CysHCl sample shows images with spatially, on average, brighter and more homogeneous emission and thereby larger QFLS values with smaller deviation, suggesting better optoelectronic properties of the films on a millimetre scale. This is expected to contribute to an improved device fill factor (FF) and open‐circuit voltage (*V*_OC_) values as discussed in the next section. Learned from our previous work,^[^ ^18^ ^]^ the improved optoelectronic property of the mixed metal Sn–Pb perovskite films prepared with the addition of amino acid salts mainly originates from the passivation effect of the ammonium functional group. Thus, we conclude that attaching additional effective base group(s) to the amino acid moiety of the ammonium salt class of additives could further improve the optoelectronic property of the mixed metal Sn–Pb perovskite films, presumably mainly owing to the reinforced passivation effect of its ammonium terminal.^[^ ^18^ ^]^

### Solar Cell Devices

**Figure 4:**
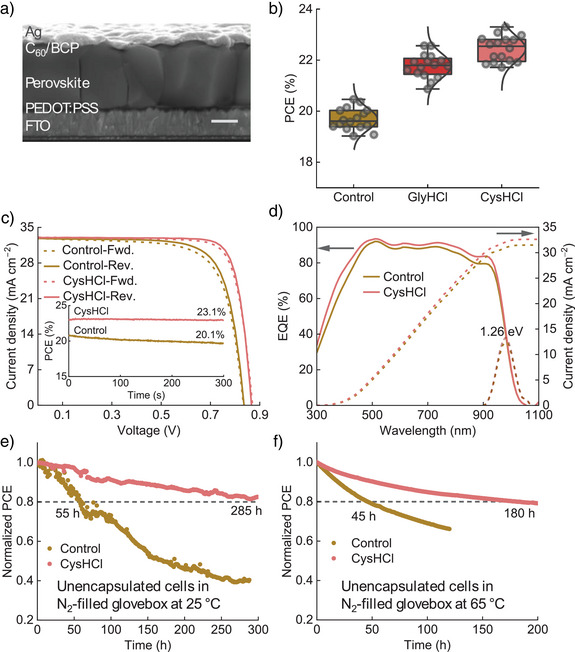
Solar cell devices. a) Cross‐sectional SEM image of the solar cells. Scale bar: 500 nm. b) Efficiency distribution of cells fabricated without an additive under study and individually with the addition of 1 mol% GlyHCl and CysHCl. c) *J*–*V* curves of the best‐performing devices. Inset: the short‐term steady‐state power output curves. d) EQE spectra and integrated *J*_SC_ curves of representative cells. −dEQE/d*λ* (dashed lines) is shown to estimate the PV bandgap of the perovskite. Normalised long‐term MPPT curves for the unencapsulated control and CysHCl devices operating under AM1.5 G in an N_2_‐filled glovebox at e) 25 °C (with initial efficiencies of 20.2% and 23.3%, respectively) and f) 65 °C (with initial efficiencies of 20.4% and 22.9%, respectively).

To further verify the improved optoelectronic properties of the films, we fabricate solar cell devices with the structure of FTO/PEDOT:PSS/perovskite/C_60_/BCP/Ag, where FTO stands for fluorine‐doped tin oxide, PEDOT:PSS for poly(3,4‐ethylenedioxythiophene) polystyrene sulfonate, and BCP for bathocuproine (Figure 4a). The designated active area of the cells is 0.0982 cm^2^. To precisely determine the best loading concentration, we fabricate the solar cells with the films deposited with the addition of 0.5, 1.0, 1.5 and 2.0 mol% of CysHCl in the same batch (Figure S12). The statistical analysis of the cell performance parameters reveals peak efficiencies at 1.0 mol% cells, with the contribution primarily coming from the improved *V*_OC_ and FF, consistent with the PLQE and QFLS results. This could be a result of the improved crystal quality and film morphology, as well as interface carrier dynamics.^[^ ^20^ ^]^ We note that the 1 mol% optimal loading amount for CysHCl is lower than that of the 2 mol% for GlyHCl we discovered previously,^[^ ^20^ ^]^ consistent with the trend of the SAXS results. We also fabricate a batch of cells to compare the effectiveness of CysHCl to GlyHCl in their 1 mol% concentration. As a result, the CysHCl cells deliver the best efficiency of 23.3% (reverse scan, *J*_SC_: 33.0 mA cm^−2^, *V*_OC_: 0.87 V, FF: 0.82), slightly higher than that of GlyHCl cells at 22.6%, but both are substantially higher than the control at 20.6% (Figure 4b). The small difference between CysHCl and GlyHCl cells is mainly attributed to the variations in *V*_OC_ and FF (Figure S13). An improvement is observed in all device parameters for cells fabricated with additives compared to the control. A steady‐state power output measurement at a fixed bias of 0.74 V for 5 min gives the efficiency of 23.1% for the CysHCl cell, higher than that of the control at 20.1% measured under 0.68 V bias for the same period (Figure 4c). We measure the external quantum efficiency (EQE) of the control and CysHCl cells, with the spectra presented in Figure 4d. The calculated −dEQE/dλ (λ = wavelength) values suggest a PV bandgap of 1.26 eV for both cells. Integrating the spectra with the standard AM1.5G spectrum generates current density values of 31.5 and 32.6 mA cm^−2^ for control and CysHCl cells, respectively. To evaluate the stability of devices, we track the evolution of the maximum power point of unencapsulated cells under simulated AM1.5G illumination in an N_2_‐filled glovebox (ISOS‐L‐1^[^ ^67^ ^]^) (Figure 4e). The CysHCl cell shows a T_80_ (the time for the device to age to 80% of its initial PCE) value of about 285 h, more than five times longer than that of the control with a T_80_ of about 55 h. In addition, we also examine the operational stability of the cells under an elevated temperature, at 65 °C, following the ISOS‐L‐2 protocol.^[^ ^67^ ^]^ We find that the CysHCl cells exhibit a T80 value of 180 h, considerably longer than the 45 h observed with the control cells (Figure 4f). The improved thermal stability can be attributed to the enhanced structural and interfacial stability of the perovskite materials and devices, likely resulting from the strong interaction provided by CysHCl for effective passivation, as well as the reduced residual DMSO (known as an oxidant of Sn^2+^ under acidic conditions and elevated temperature^[^ ^68^, ^69^ ^]^) in the modified films. These results thus suggest that the multifunctional additive designed here could concurrently improve solar cell efficiency and operational lifetime, with a reasonably low consumption of the additive materials.

## Conclusions

In summary, we have identified the different fundamental chemical characteristics of Sn^2+^ and Pb^2+^ cations from the perspective of HSAB principle and the series of implications in mixed‐metal Sn–Pb perovskite precursor solutions. The differences in hard and soft acidity between the two metals offer the possibility to introduce nucleophilic species of varying hard–soft basicity for establishing desirable interactions with each of the metals. Thus, we propose to enhance the simultaneous access of the harder Sn^2+^ with the hard base carboxyl and the softer Pb^2+^ with the soft base thiol. To study this system, we used CysHCl to prove the preference of each of the groups towards the metals and their mechanistic implications in this mixed metal system. In particular, the increased control over both metal species by functional groups from the same molecule shows the ability to manipulate the colloidal properties of perovskite precursors in solution and thus contribute to the following crystallisation process. In addition, the thiol group attached to the amino acid moiety reinforces the passivation effect of the ammonium group, leading to further improved optoelectronic properties of the films, giving the best QFLS values of up to 0.95 eV, which is 96% of the radiative limit of a 1.26 eV bandgap material. As a result, the mixed‐metal Sn–Pb PSCs showed the best efficiency values of up to 23.3% and improved operational stability, with a T_80_ of over 280 and 180 h at 25 and 65 °C, respectively. Furthermore, these results point out the critical role played by the inorganic component in the controlled crystallisation of mixed Sn–Pb perovskite thin films, suggesting the presence of inorganic‐based “intermediates” during the process. Overall, this study provides straightforward chemical guidelines to desirably control the environment of metal‐centred species in halide perovskite materials, contributing to a well‐controlled solution processing of thin films for optoelectronic devices. We also anticipate more new experimental findings that are guided by or aligned with clear fundamental principles, which should accelerate the construction of datasets and data augmentation to advance artificial intelligence‐assisted, data‐driven research in the scientific domain shortly.

## Author Contributions

S.H. conceived the idea and prepared the initial draft of the manuscript. S.H. and J.P. designed the experiments. S.H. performed the SEM, UV–vis absorption, XRD, and PLQE measurements. S.H., W.L., J.P., and A.D. performed the NMR characterisations and captured the solution pictures. S.H. (in Japan and UK) and X.S. (in China) fabricated the cells, performed the related characterisations, and conducted the XPS measurements. L.G. and F.D.A. performed the theoretical calculations. P.Z. discussed the theoretical results and helped to edit the text. A.D. conducted the PL image measurements. S.H.T.C. collected grain size. Y.I. assisted with the SEM measurements. R.M. assisted with the AFM measurements. F.Y., M.A., M.A.F., A.H., S.S., S.A., and A.A. provided the SAXS results. J.P., L.G., G.L., S.F., M.L., S.Y., A.A., A.W., F.D.A., and H.J.S. contributed to the revision of the manuscript. M.L., A.A., A.W., F.D.A., and H.J.S. supervised the project and raised the grant funding for the laboratory infrastructure and research. All authors commented on and contributed to the revision of the written manuscript.

## Conflict of Interests

H.J.S. is the co‐founder and CSO of Oxford PV Ltd. A.W. is the co‐founder and CSO of EneCoat Technologies Co., Ltd.

## Supporting information



Supplementary Information

## Data Availability

The data supporting the findings of this study are available from the corresponding author upon reasonable request.
