# Supplementary material for: Accessing Metal‐Containing Species in Tin–Lead Perovskite Precursor Solutions via Molecular Strategies Guided by the Hard–Soft Acid–Base Principle
Source: Angew Chem Int Ed Engl. 2025 Aug 19;64(41):e202514010. doi: 10.1002/anie.202514010 (PMC12501668; doi:10.1002/anie.202514010)
Supplement: Supplementary file 1 — Supplementary Information [file ANIE-64-e202514010-s001.pdf]

Supporting information

Accessing Metal-Containing Species in Tin–Lead Perovskite Precursor Solutions via Molecular Strategies Guided by the Hard–Soft Acid-Base Principle

Shuaifeng Hu[1,†,*], Xinru Sun[2,†], Wentao Liu[3,†], Luca Gregori[4], Pei Zhao[5], Jorge Pascual[6,*], André Dallmann[7], Akash Dasgupta[1], Fengjiu Yang[3], Guixiang Li[8], Mahmoud Aldamasy[3], Silver-Hamill Turren-Cruz[9], Marion A. Flatken[3], Sheng Fu[10], Yasuko Iwasaki[11], Richard Murdey[11], Armin Hoell[3], Susan Schorr[3], Steve Albrecht[3], Shangfeng Yang[12], Antonio Abate[3,13], Atsushi Wakamiya[11], Filippo De Angelis[4,14], Meng Li[2,*], and Henry J. Snaith[1,*]

[1]Clarendon Laboratory, Department of Physics, University of Oxford, Oxford OX1 3PU, U.K.

[2]Key Laboratory for Special Functional Materials of Ministry of Education, School of Nanoscience and Materials Engineering, Henan University, Kaifeng, 475004, China

[3]Helmholtz-Zentrum Berlin für Materialien und Energie GmbH, Hahn-Meitner-Platz 1, 14109 Berlin, Germany.

[4]Department of Chemistry, Biology and Biotechnology, University of Perugia, Via Elce di Sotto 8, 06123, Perugia, Italy.

[5]Research Center for Computational Science, Institute for Molecular Science, Okazaki, 444-8585, Japan.

[6]Polymat, University of the Basque Country UPV/EHU, 20018 Donostia-San Sebastian, Spain.

[7]Institut für Chemie, Humboldt-Universität zu Berlin, Berlin, Germany.

[8]School of Materials Science and Engineering, Southeast University, Nanjing, 211189, Jiangsu, China.

[9]Instituto de Ciencia de los Materiales (ICMUV), Universitat de Valencia, 46980 Paterna, Spain.

[10]School of Physics and Electronic Science, Engineering Research Center of Nanophotonics and Advanced Instrument Ministry of Education, East China Normal University, Shanghai 200062, China.

[11]Institute for Chemical Research, Kyoto University, Gokasho, Uji, Kyoto 611-0011, Japan.

[12]Key Laboratory of Materials for Energy Conversion, Anhui Laboratory of Advanced Photon Science and Technology, Department of Materials Science and Engineering, University of Science and Technology of China, Hefei, Anhui 230026, P. R. China

[13]Department of Chemical, Materials and Production Engineering, University of Naples Federico II, Piazzale Tecchio 80, 80125 Fuorigrotta, Naples, Italy.

[14]SKKU Institute of Energy Science and Technology (SIEST), Sungkyunkwan University, Suwon 440-746, South Korea.

[†]These authors contributed equally.

Keywords: perovskite • photovoltaics • metal centre • solution chemistry • Lewis acid and base • crystallisation

Experimental Section

Materials

For experiments in Kyoto and Oxford: Unless otherwise stated, all materials were used as received without further purification. Methylammonium iodide (MAI, >99.0%) and formamidinium iodide (FAI, >98.0%) were purchased from Greatcell Solar Materials. Bathocuproine (BCP, >99.0%), lead iodide (PbI_2, 99.99%, trace metals basis), cysteine hydrochloride, and 2-Mercapto-ethyl-amine hydrochloride were purchased from Tokyo Chemical Industry Co., Ltd. (TCI). Caesium iodide (CsI, 99.999%, metals basis) was purchased from Alfa Aesar. Ammonium thiocyanate (NH_4SCN, 99.99% trace metals basis), tin fluoride (SnF_2, 99%), tin iodide (SnI_2, beads, 99.99%, trace metals basis), glycine hydrochloride (GlyHCl, ≥99%), and poly(methyl methacrylate) (PMMA, >98%) were purchased from Sigma-Aldrich Co., Ltd. (Sigma-Aldrich). Poly(3,4-ethylenedioxythiophene):poly(styrene sulfonate) (PEDOT:PSS) aqueous solution (Clevios PVP AI 4083) was purchased from Heraeus Co., Ltd. Fullerene C_{60} (sublimed, 99.99%) was purchased from ATR Company. Dehydrated dimethylsulfoxide (DMSO, super dehydrated) and isopropanol (IPA, super dehydrated) were purchased from FUJIFILM Wako Pure Chemical Co., Ltd or Sigma-Aldrich Co., Ltd. (Sigma-Aldrich). Dehydrated *N,N*-dimethylformamide (DMF), and chlorobenzene were purchased from Kanto Chemical. Co., Inc. or Sigma-Aldrich Co., Ltd. (Sigma-Aldrich). All of these solvents were degassed by Ar gas bubbling for 1 h and further dried with molecular sieves (3 Å) in an

Ar- or N_2-filled glove box (H_2O, O_2 < 0.1 ppm) before use in Japan. No particular treatment was applied for the solvent used in the UK.

For experiments in Henan: Tin iodide (SnI_2, 99.999%), tin fluoride (SnF_2, 99.999%), lead iodide (PbI_2 99.999%), ammonium thiocyanate (NH_4SCN, 99.99%), F-doped tin oxide (FTO), fullerene (C_{60}, 99.9%), bathocuproine (BCP, 99.9%), L-Cysteine monohydrochloride (CysHCl, 99%), glycine hydrochloride (GlyHCl, 99%) were purchased from Libra Technology Corporation. Caesium iodide (CsI, 99.999%) was purchased from Xi'an Yuri Solar Co., Ltd. Formamidinium iodide (FAI, 99.99%), methylammonium iodide (MAI, 99.99%) were purchased from GreatCell Solar (Australia). Chlorobenzene (CB, 99.8%, SuperDry, with molecular sieves), isopropanol (IPA, 99.5%, SuperDry, with molecular sieves), ethanol and dimethyl sulfoxide (DMSO, 99.7%, SuperDry, with molecular sieves), *N*,*N*-dimethylformamide (DMF, 99.8%, SuperDry, with molecular sieves), were purchased from J&K scientific. Poly(3,4-ethylenedioxythiophene):poly(styrene sulfonate) dry re-dispersible pellets (PEDOT:PSS(4083)) were purchased from Xi'an Polymer Light Technology Corp. Ethanol (99.7%) was purchased from Amethyst Chemicals. Cysteamine hydrochloride (98%) was purchased from Macklin.

Fabrication of perovskite thin films

1.26 eV-bandgap mixed tin–lead perovskites

The perovskite film was prepared in an Ar- (in Japan only) or N_2-filled glove box (H_2O, O_2 < 0.1 ppm). The 1.8 M $Cs_{0.1}FA_{0.6}MA_{0.3}Sn_{0.5}Pb_{0.5}I_3$ perovskite precursor solution was prepared by mixing CsI, FAI, MAI, SnI_2, PbI_2, SnF_2, and NH_4SCN at projected quantities in a solvent mixture of DMSO and DMF (1:3, v:v) for the control samples. For the CysHCl samples, 0.5, 1.0, 1.5 and 2.0 mol% of CysHCl was added to the control solution. For the samples containing the different amino acids and derivatives, 1.0 mol% of the corresponding compound was added to the control solution. The precursor solutions were stirred at 45 °C for about 40 min and filtered through a 0.20 μm PTFE filter before use. To spin coat the films, 200 μL of the room temperature precursor solution was applied to the substrate. A two-step spin coating program was used. The first step was 1000 rpm for 10 s with an acceleration of 200 rpm s^{-1}, and the second was 4000 rpm for 40 s with a ramp-up of 1000 rpm s^{-1}. The 300 μL chlorobenzene antisolvent was quickly dripped onto the surface of the spinning substrate over an interval of 1 s during the second spin coating step, 20 seconds before the end of the procedure. The substrate was then immediately annealed on a 100 °C hot plate for 10 min, followed

by annealing at 65 °C for over 10 min to avoid glovebox vapour ingress of the as-prepared films, then the films were cooled down to room temperature for the following processes. For the $EDAI_2$ post-treatment, 1.0 mg $EDAI_2$ was added to 1.0 mL IPA and 1.0 mL chlorobenzene[1]. The spin coating process was set as 4000 rpm for 20 s with an acceleration of 1333 rpm s^{-1}. Following spin coating, the films were immediately annealed again at 100 °C for around 5 min.

Fabrication of solar cell devices

Single-junction mixed tin–lead perovskite solar cells

F-doped tin oxide (FTO) substrates (2.5 cm by 2.5 cm, 10 Ω sq^{-1}, Libra Technology Corporation)were consecutively cleaned with a 15 min ultrasonic bath in water, acetone, and detergent solution, water, and ethanol, followed by drying with an air gun, and UV/ozone for 30 min treatment. The PEDOT:PSS hole transport layer was fabricated from an aqueous dispersion (without diluting), which was filtered through a 0.45 μm PVDF filter and then spin-coated on the FTO substrate using a spin program of 10 s at 500 rpm followed by 30 s at 4000 rpm. The films were then annealed in air at 140 °C for 15 min. After transferring to an N_2-filled glove box (H_2O, O_2 < 0.1 ppm), the substrates were degassed at 140 °C for 10 min. The samples were moved under N_2 to a vacuum deposition chamber, where 40 nm of C_{60} (deposition rate 0.03-0.05 nm s^{-1}) and 12 nm of BCP (deposition rate 0.01-0.03 nm s^{-1}) were deposited by thermal evaporation. The top electrode was prepared by depositing 110 nm of silver (Ag) through a shadow mask. The deposition rate for Ag was first set as 0.005 nm s^{-1} to reach 5 nm, then raised to 0.01 nm s^{-1} to reach 20 nm, and finally raised to 0.08 nm s^{-1} to reach the target thickness. For the devices fabricated at Oxford or Kyoto, the patterned glass/FTO substrates (15 Ω sq^{-1}, 15 Ω sq^{-1} Latech Scientific Supply Pte. Ltd. or 10 Ω sq^{-1}, AGC Inc.) were used. The PEDOT:PSS hole transport layer was fabricated from an aqueous dispersion (without diluting), which was filtered through a 0.45 μm PVDF filter and then spin-coated on the FTO substrate using a spin program of 10 s at 500 rpm, followed by 30 s at 4000 rpm. The films were then annealed in air at 140 °C for 20 min. After transferring to an Ar- or N_2-filled glove box (H_2O, O_2 < 0.1 ppm), the substrates were degassed at 140 °C for 30 min. The perovskite layer was fabricated on PEDOT:PSS following the above-mentioned procedure. The samples were moved under Ar or N_2 to a vacuum deposition chamber, where 20 nm of C_{60} (deposition rate 0.01 nm s^{-1}) and 8 nm of BCP (deposition rate 0.01 nm s^{-1}) were deposited by thermal evaporation. The top electrode was prepared by depositing 100 nm of silver (Ag) through a shadow mask with the procedure above.

Characterizations

Photoluminescence quantum efficiency measurements

Photoluminescence quantum efficiency (PLQE) of samples was determined according to the method of de Mello et al.[2] Samples were placed inside an integrating sphere and excited from the substrate side with a 657 nm continuous wave laser excitation source (Thorlabs) at 70.9 mW cm^{-2} (equivalent to one sun for a 1.25 eV bandgap) with a large spot size of 0.15 cm^2. The resulting PL signal was collected via a fibre bundle (Ocean Optics QR600 7 SR125BX) coupled with a spectrometer (QE Pro, Ocean Optics). Three different spots were measured on each substrate.

Photoluminescence (PL) emission spectra with a signal-to-noise ratio below 2 were not used, which corresponded to a noise floor of around 0.03% PLQE. Additionally, the detector used was not able to effectively detect PL above 1050 nm, thus leading to a slight underestimation.

QFLS (Quasi-Fermi level splitting) was calculated according to the following equation[3]:

$$QFLS = QFLS_{rad} + k_B T \ln(PLQE)$$

The QFLS images were obtained using the same setup as our previous report[4], which is also demonstrated with the scheme below. The sample was optically excited with a 450nm LED and electronically contacted by a source meter.

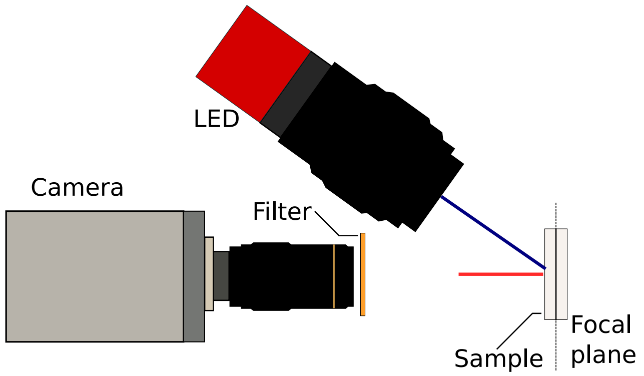

The intensity of the LED, which corresponds to an equivalent 1 sun illumination, was determined by measuring the current on a fully fabricated device at a short circuit and varying the intensity till this current matched that measured on our calibrated solar simulator. The PL images were captured on an ANDOR ZYLA CMOS image sensor. A long pass filter was used to stop the excitation light.

The PLQE was estimated by imaging the excitation light incident on a white reference plate, placed on the focal plane of the camera, without a filter. We use this as a dividing factor for the PLQE, taking into account the wavelength-dependent response of the camera lenses and the image sensor itself. The QFLS maps may be obtained from the PLQE ma via the relation:

$$QFLS = QFLS_{rad} + kT\ln(PLQE).$$

For each measurement, the illumination was held for 30 seconds before measurement, to allow for the PL to stabilise.

Scanning electron microscopy (SEM): SEM at Kyoto was performed with a Hitachi S8010 ultra-high-resolution scanning electron microscope (Hitachi High-Tech Corporation) under the accelerating voltage of 2 kV for the experiments (cross and top view of the perovskite films) conducted in Kyoto. The cross-section images of the devices were recorded by a FEI Quanta 3D FEG microscope at Oxford. A 10-kV electron beam with a spot size of 2 and a secondary electron detector was used. A typical working distance was about 10 mm, and dwell time for single-pass image acquisition was 5 μs. Focusing and alignment were done away from imaged areas to minimise electron-beam-induced damage.

UV–vis absorption measurement was performed with a Shimadzu UV-3600 plus spectrometer (Shimadzu Co., Ltd.).

Photocurrent–voltage (*J–V*) curves were measured in an N_2-filled glove box (H_2O, O_2 <0.1 ppm) using a performed sunlight simulator with a digital source meter (IVX-50, EnLi Technology, Taiwan). The light intensity of the illumination source was calibrated using a standard silicon solar cell. Each device was measured with a 10-mV voltage step and a 100 mW/cm^2 illumination intensity. The device's active area was defined by an optical mask, 0.0982 cm^2 for the regular devices. Steady-state power output (SPO) measurement was performed by holding the device at the voltage of the maximum power point, as determined by J–V characterisation, and monitoring the current density over the course of 300 s.

External quantum efficiency (EQE) spectra were measured using a QE-R EQE system (EnLi Technology, Taiwan). The incident photon-to-current conversion efficiency (IPCE) was determined, and the incident light intensity was calibrated with a standard SiPD S1337-1010BQ silicon photodiode.

Long-term stability tests: Operational stability tests were performed using a stability test instrument purchased from Suzhou Derui Keyi Instrument and Equipment Co., Ltd. (PVLT-6001M-64D). Unencapsulated devices were subjected to continuous light illumination in a N_2-filled glovebox, and their maximum power output was tracked under 25 and 65 °C. The device temperature was controlled by a temperature sensor and thermistor coupled in the aluminium plate of the device slot (see picture below).

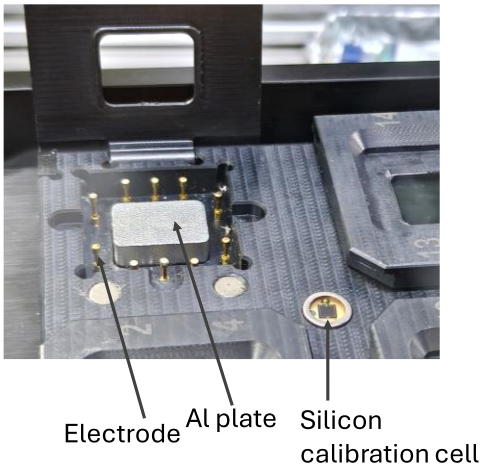

X-ray photoelectron spectroscopy (XPS) was performed on different perovskite bottom films were performed with a photoelectron spectrometer (Thermo SCIENTIFIC ESCALAB 250Xi) with a monochromatized X-ray source (Al Ka). In Japan, X-ray photoelectron spectroscopy (XPS) was performed with a photoelectron spectroscopy system (PHI 5000 Versa Probe II, ULVAC-PHI). Monochromated Al Kα (1486.6 eV) radiation with an operating power of 50 W (15 kV voltage) was used in all the XPS measurements. The diameter of the analysed area was 200 μm. The take-off angle was 45° to the substrate.

XRD measurements were performed on a Rigaku SmartLab equipped with a goniometer-mounted 2D hybrid pixel array detector (HyPix-3000) with a rotating Cu Kα source (λ = 1.5406 Å). Perovskite films were deposited on top of PEDOT:PSS with glass/FTO as substrates to mimic the growth conditions in full devices, and covered with a thin film of spin-coated PMMA to prevent direct exposure to air.

Atomic force microscopy (AFM) measurements were performed with a Picoscan Plus AFM instrument used in AC mode with Nanoworld NCST probes.

^{1}H NMR spectra were recorded on a Bruker Avance-400 spectrometer (400 MHz). The NMR chemical shifts are reported in ppm relative to the residual protons of DMF-d_7 (δ = 7.95 ppm of –HC=O proton in ^{1}H NMR) and/or DMSO-d_6 (δ = 2.54 ppm in ^{1}H NMR).

Metal NMR: The 0.5 M PbI_2, SnI_2, $CsSnI_3$, and $CsPbI_3$ together with 1.0 M CsI and $CsPb_{0.5}Sn_{0.5}I_3$ solutions were prepared in a solvent mixture of DMF-d_7 and DMSO-d_6 (DMF-d_7:DMSO-d_6 = 3:1 (v/v)). 0.5 mg ml^{-1} Sn(0) powder was added to the Sn-containing solutions to scavenge the potential Sn(IV) introduced by the oxidation of Sn(II). As stated in the perovskite preparation section, the related solutions were stirred at 45 °C for about 30 min. All the solutions were divided into 4 vials equally. For the CysHCl, GlyHCl, and MeAHCl samples, 5 mol% of CysHCl, GlyHCl, and MeAHCl was added and then stirred at 45 °C for another 30 min. The related solutions were then filtered through a 0.20-μm PTFE filter before use. 600 μL solutions were added to clean NMR tubes for measurement.

The spectra were all acquired on a Bruker AV4 600 MHz equipped with a room-temperature TBO or BBO probe head. Typically, for ^{119}Sn, ^{207}Pb and ^{133}Cs sweep widths of 507.88 ppm, 903.66 ppm, and 1443.66 ppm were used, and 64k, 16k, and 16k points were acquired, respectively. The centre frequency had to be adjusted from sample to sample in order to detect the desired signal; therefore, on new samples, a full scan of the possible shift range for these samples was usually acquired (typically for ^{119}Sn and ^{207}Pb between 2000 and −2500 ppm). We used a 30° pulse to minimise the recycle delay to 1 s, 0.2 s, and 0.2 s for 119Sn, 207Pb, and 133Cs, respectively. All spectra were measured proton decoupled. The number of scans thus ranged from 1024 for ^{119}Sn and typically 2048 scans (and for diluted samples up to 128k scans) for ^{207}Pb and ^{133}Cs.

Small-angle X-ray scattering (SAXS) measurements were performed using synchrotron radiation at the four-crystal monochromator X-ray beamline in the laboratory of PTB (Physikalisch-Technische Bundesanstalt) at BESSY-II[5]. The SAXS instrument of Helmholtz-Zentrum Berlin (HZB) contains an adjustable about 3 m long support structure with a long edge-welded bellow system to enable the changing of the sample to detector distance[6]. The 2D scattering images were collected by a windowless 1M PILATUS2 in-vacuum hybrid-pixel detector, produced by Dectris. The measurements were carried out at two different distances (0.8 and 3.7 m) at photon energies of 10 keV and 8 keV, respectively. X-ray energies and sample-to-detector distances are chosen to get the maximum possible q-range, and the X-ray energies are chosen with respect to the low sample transmissions. Thus, a q-range from 0.05 to 8.5 nm^{-1} was covered (size range of 125.6 to 0.74 nm in real space). The precursor solutions were measured with an acquisition time of 600 s with three repetitions for the

short distance and long distance to achieve good quality data and to monitor unwanted but possible changes of the specimen over time (which can therefore be safely excluded). Due to the low transmittance of the lead-containing precursor solutions, especially thin (0.1 mm), rectangular borosilicate cuvettes (with a wall thickness of 0.1 mm) purchased from CM Scientific, UK, were used. Using a low-scatter pinhole of germanium 500 μm in diameter, the total flux on the sample has been determined for each energy. For the long distance (3.7 m) at 8 keV, we indicated 4.8×10^9 and 2.6×10^9 photons/s for the short distance (0.8 m) at 10 keV. Data reduction and radial averaging & fitting: For data reduction as well as for the radial averaging to the 1D scattering pattern, the BerSAS software was used, an advanced version of the BerSAS software[7] applicable for SAXS and SANS. All SAXS curves of the investigated samples were fitted with the program SASfit[8]. In order to get a general idea of the order of magnitude, a structure model that includes a spherical form factor and a hard-sphere structure factor was chosen to fit all sample scattering curves. The herein investigated particles were considered as hard spheres. Thereby, these particles are assumed as incompressible, resulting in fixed radii for each particle and an infinite repulsive force at a certain interparticular separation. The hard spheres model neglects attractive forces but describes fairly well a various number of colloids in organic solvents[9-11]. We used the monodisperse Percus-Yevick approximation for hard spheres to fit the interaction of the analysed colloidal particles[12,13]. All the solutions were filtered using a PTFE 0.20-μm filter before being subjected to the capillary for measurements.

Computational methods

DFT calculations on isolated complex/molecule interactions are carried out using Gaussian09[14] software. In order to predict the relative equilibrium geometries and the adsorption energy in solution, we use the LANL2DZ basis to set for heavy Pb and I atoms together with the LANL2 pseudopotentials for the core electrons, whereas, for light atoms (C, H, N, O, S), we employ the 6-31G* basis sets with B3LYP exchange-correlation functional[15]. The solvation models used in the present work are simulated using both implicit solvation models (Polarised Continuum Model, PCMs in Gaussian09) and the addition of explicit coordination molecules necessary for the particular chemical interactions of interest. Solid-state calculations are carried out in the supercell approach using the CP2K software package[16]. The Perdew–Burke–Ernzerhof (PBE) exchange-correlation functional[17] with DFT-D3 Van der Waals corrections[18], norm-conserving Goedecker-Teter-Hutter pseudopotentials and DZVP Gaussian basis set[19] are used. Calculations are carried out at the Γ point in the Brillouin zone (BZ). The different adsorption orientations for the cysteine salt and molecule are

simulated in the 2×2 in-plane slab model of $MAPb_{0.5}Sn_{0.5}I_3$, created starting from the tetragonal phase of $MAPb_{0.5}Sn_{0.5}I_3$, by keeping cell parameters fixed at experimental values[20]. In all cases, 20 Å of vacuum have been added in the non-periodic direction perpendicular to the slabs to avoid interaction with the replica.

The binding energy E_{Bin} for each configuration is calculated as follows:

$$E_{\mathrm{Bin}} = E_{\mathrm{Slab+Mol}} - (E_{\mathrm{Slab}} + \mathrm{E}_{\mathrm{Mol}})$$

Where $E_{\mathrm{Slab+Mol}}$ is the total energy of the slab and the adsorbed cysteine salt. The more negative the binding energy, the more stable the adsorption configuration.

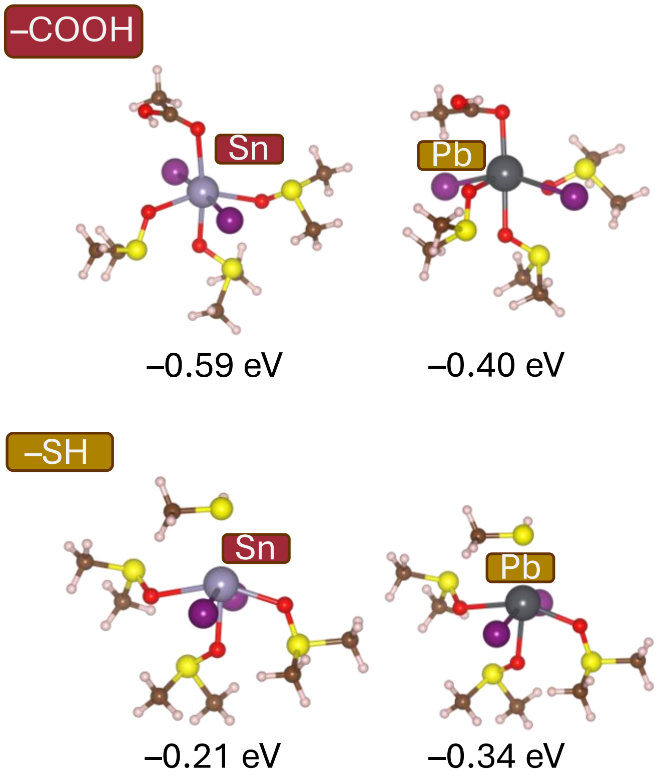

Figure S1. DFT-optimised configurations of CH_3COOH/CH_3SH and SnI_2•$(DMSO)_3$/PbI_2•$(DMSO)_3$ complexes with –COOH or –SH binding to the metal centres, with the binding energies provided.

We further investigate the binding behaviour of the complete cysteine molecule on the surface of a mixed Sn–Pb perovskite ($MAPb_{0.5}Sn_{0.5}I_3$) considering two adsorption modes, one in which the thiol binds to Pb(II) and the carboxyl binds to Sn(II) (Sn–O/Pb–S), and a second one with the binding sites reversed (Sn–S/Pb–O). These adsorption modes are analysed in terms of binding energy, bond lengths, and structural distortions upon adsorption. The Sn–O/Pb–S adsorption mode, where the sulfur atom of the thiol forms a covalent bond with the Pb atom and the oxygen atom of the carboxyl coordinates with Sn, appears to be the most energetically stable configuration with a binding energy of –0.90 eV, lower than the –0.83 eV of the Sn–S/Pb–O configuration (**Figure S2**). In addition, minor structural distortions are observed on the cysteine molecule and the perovskite surface upon adsorption. We calculated bond lengths of 2.97 and 2.91 Å for Pb–S and Sn–O, respectively, indicating a moderately strong interaction between the cysteine functional groups and the perovskite surface atoms with minimal perturbation of the lattice structure. On the other hand, in Sn–S/Pb–O adsorption model, the bond lengths for the Pb–O and Sn–S are 3.12 and 2.90 Å, respectively, together with a larger structural distortion compared to the Sn–O/Pb–S configuration, which might be one of the factors contributing to the high calculated binding energy. Based on binding energies and structural analyses, we conclude that the thiol –SH and carboxyl –COOH groups energetically prefer to bind to Pb(II) and Sn(II) sites, respectively, of the mixed Sn–Pb perovskites. This could be attributed to favourable electrostatic interactions between the oxygen lone pairs and Sn(II), which surpass those with Pb(II). These results align with the chemical affinity evaluation, agreeing with the HSAB characteristics of the species.

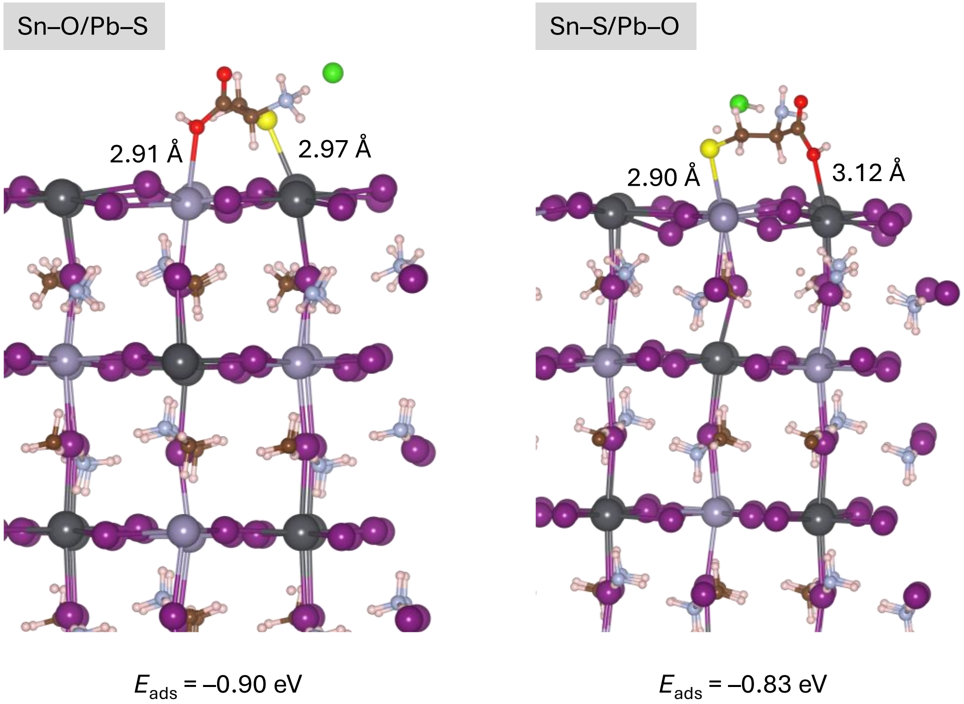

Figure S2. DFT-optimised Sn–O/Pb–S and Sn–S/Pb–O configurations of the CysHCl at the metal-terminated surfaces of a $MASn_{0.5}Pb_{0.5}I_3$ perovskite lattice, with the bond length and adsorption energy values provided.

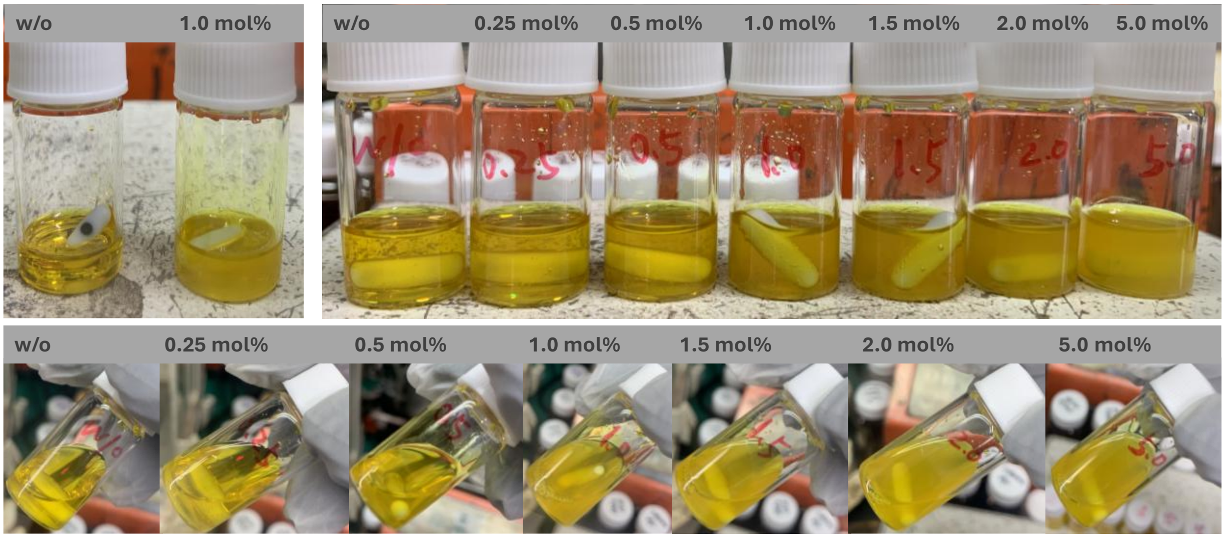

Figure S3. Photographs of the mixed Sn–Pb perovskite precursor solutions, 1.8 M $Cs_{0.1}FA_{0.6}MA_{0.3}Sn_{0.5}Pb_{0.5}I_3$ with 5 mol% of SnF_2 and 2 mol% of NH_4SCN, prepared without and with the addition of 0.25, 0.5, 1.0, 1.5, 2.0, and 5.0 mol% of the cysteine hydrochloride, were captured after the preparation of about one hour. The top left pictures were taken for the first time when the phenomena were observed.

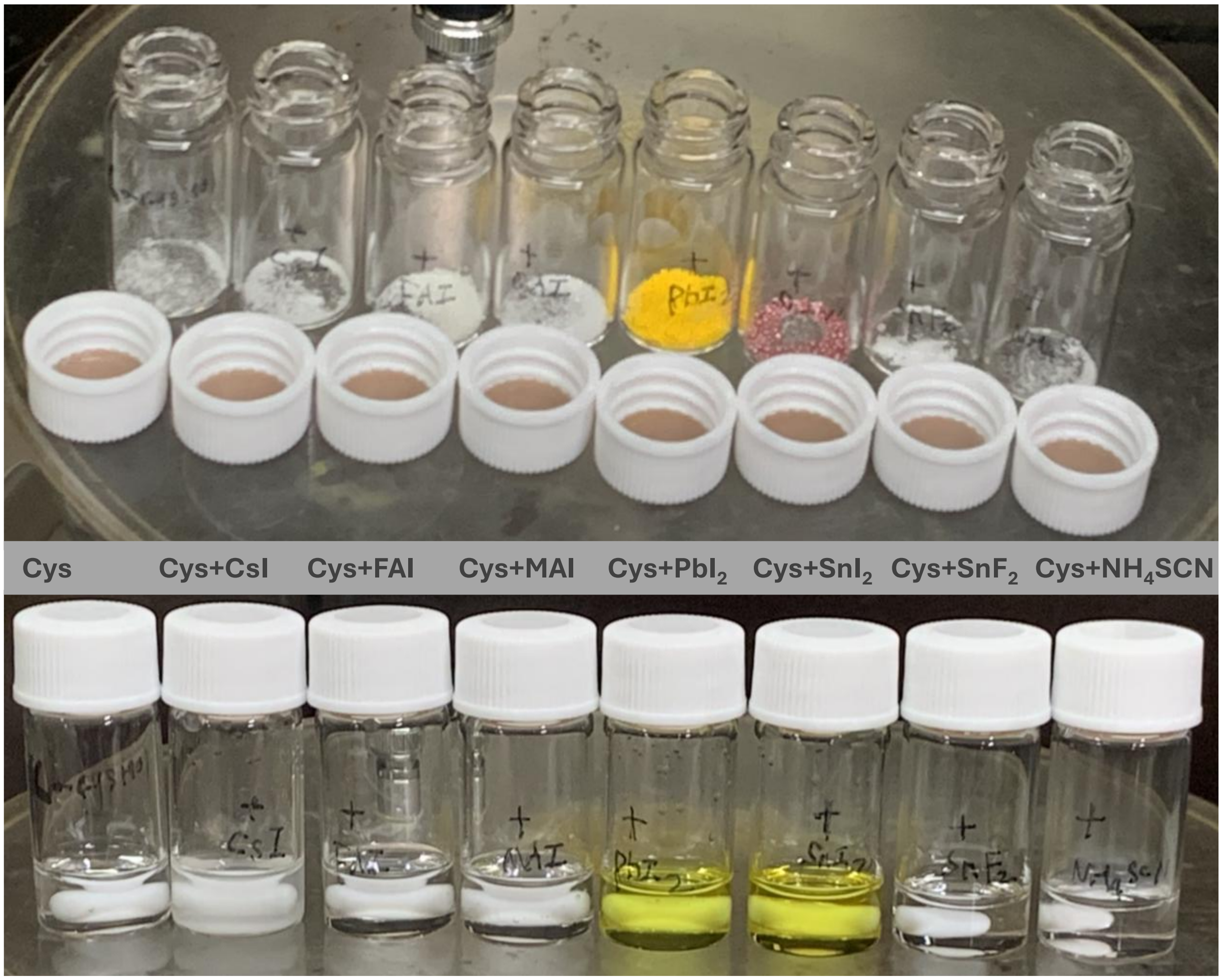

Figure S4. Photographs of the cysteine hydrochloride and cysteine hydrochloride with perovskite precursor materials (top), and their dissolved solutions, in 3:1, v:v, DMF:DMSO (bottom).

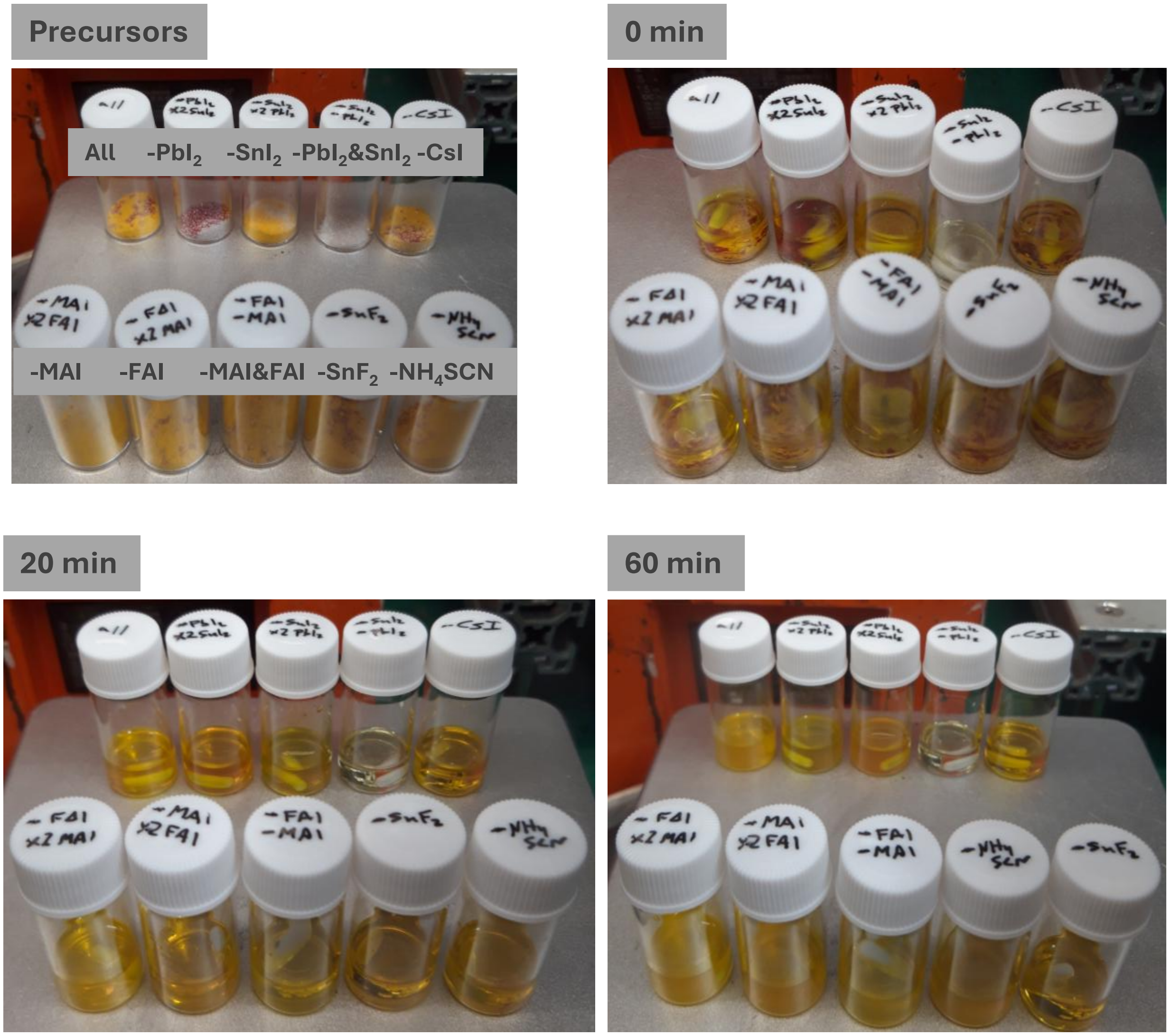

Figure S5. Photographs of the precursors with the addition of 1.0 mol% of CysHCl, presenting cases of full composition (All, $Cs_{0.1}FA_{0.6}MA_{0.3}Sn_{0.5}Pb_{0.5}I_3$ with 5 mol% of SnF_2 and 2 mol% of NH_4SCN), the amount of PbI_2 replaced with SnI_2 (-PbI_2), the amount of SnI_2 replaced with PbI_2 (-SnI_2), no B-site metal sources (-PbI_2&SnI_2), no CsI (-CsI), the amount of MAI replaced with FAI (-MAI), the amount of FAI replaced with MAI (-FAI), no organic A-site sources (-MAI&FAI), no SnF_2, and no NH_4SCN. The picture of the corresponding solutions is captured after stirring at 45 °C for 0, 20, and 60 minutes.

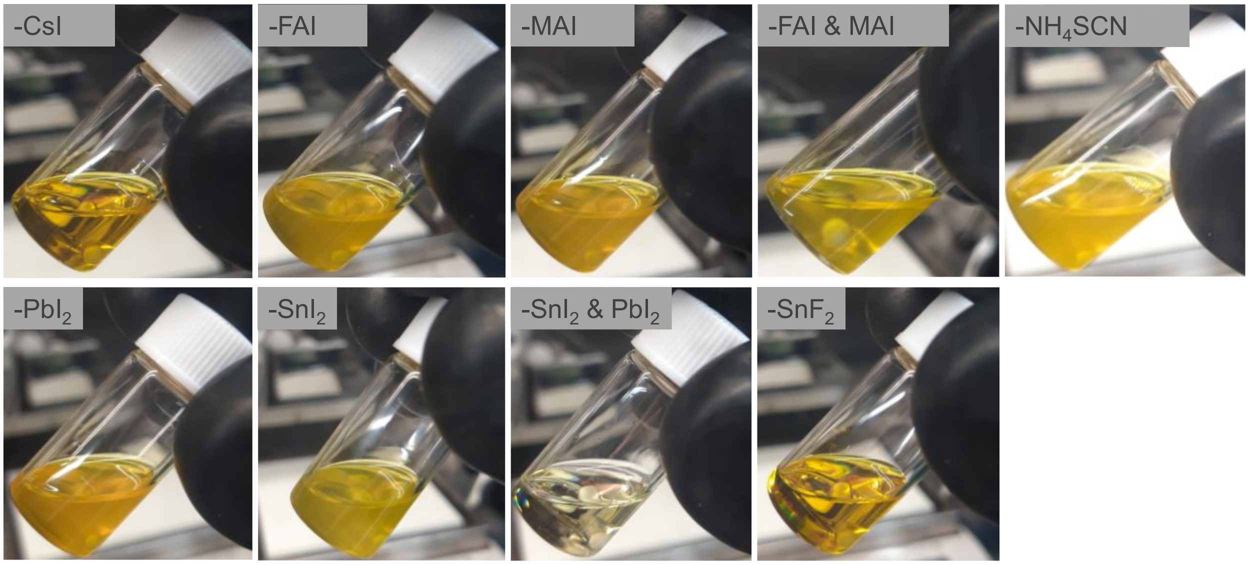

Figure S6. Photographs of the precursors with the addition of 1.0 mol% of CysHCl, presenting cases of compositions with no CsI (-CsI), the amount of MAI replaced with FAI (-MAI), the amount of FAI replaced with MAI (-FAI), no organic A-site sources (-MAI&FAI), no NH_4SCN, the amount of PbI_2 replaced with SnI_2 (-PbI_2), the amount of SnI_2 replaced with PbI_2 (-SnI_2), no B-site metal sources (-PbI_2&SnI_2), and no SnF_2. The picture of the corresponding solutions is captured after stirring at 45 °C for 60 minutes.

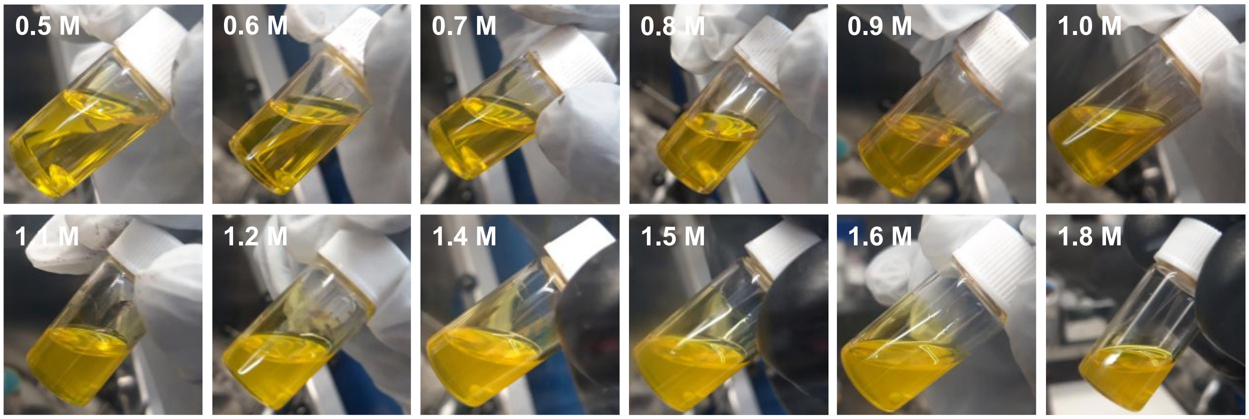

Figure S7. Photographs of the full perovskite precursor solutions with the addition of 1.0 mol% of CysHCl diluted from a concentration of 1.8 M down to 0.5 M.

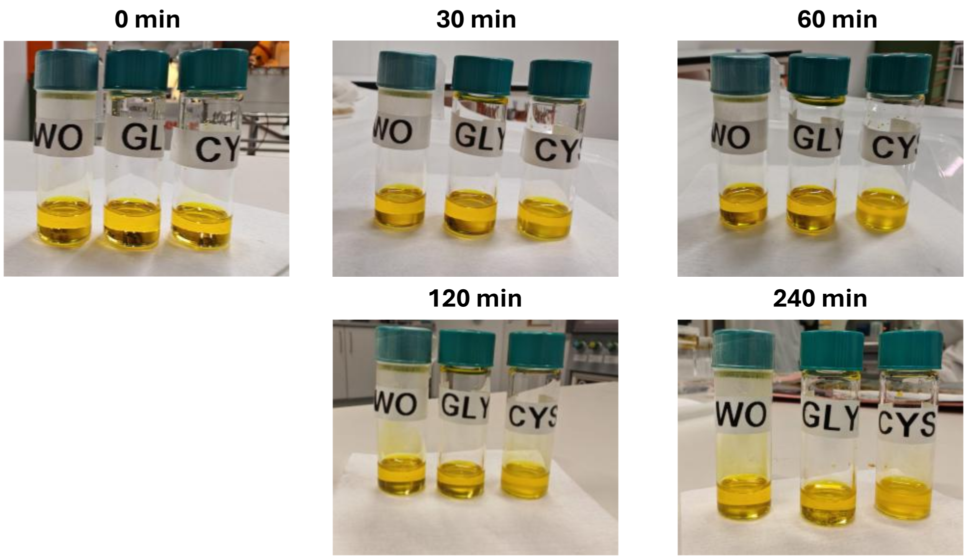

Figure S8. Photographs of the mixed Sn–Pb perovskite precursor solutions, 1.8 M $Cs_{0.1}FA_{0.6}MA_{0.3}Sn_{0.5}Pb_{0.5}I_3$ with 5 mol% of SnF_2 and 2 mol% of NH_4SCN, prepared without (WO/Control) and with the addition of 2.0 mol% of GlyHCl (GLY) and CysHCl (CYS), are captured after the filtration of the solutions for 0, 30, 60, 120, and 240 min, respectively.

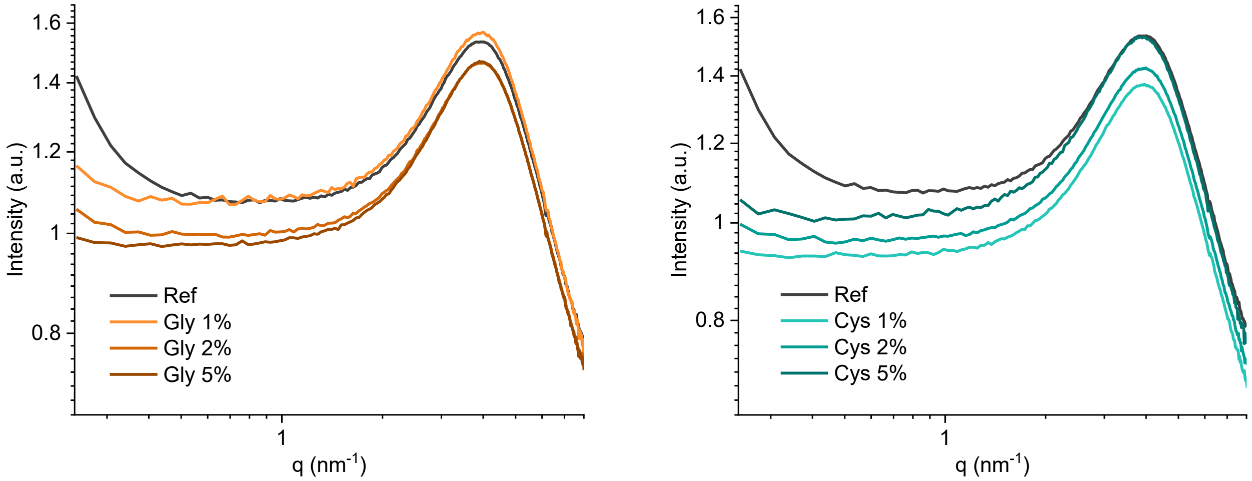

Figure S9. SAXS scattering curves of the mixed Sn–Pb perovskite precursor solutions (1.8 M $Cs_{0.1}FA_{0.6}MA_{0.3}Sn_{0.5}Pb_{0.5}I_3$ with 5 mol% SnF_2 and 2 mol% NH_4SCN) prepared with the addition of 1, 2, and 5 mol% GlyHCl and CysHCl.

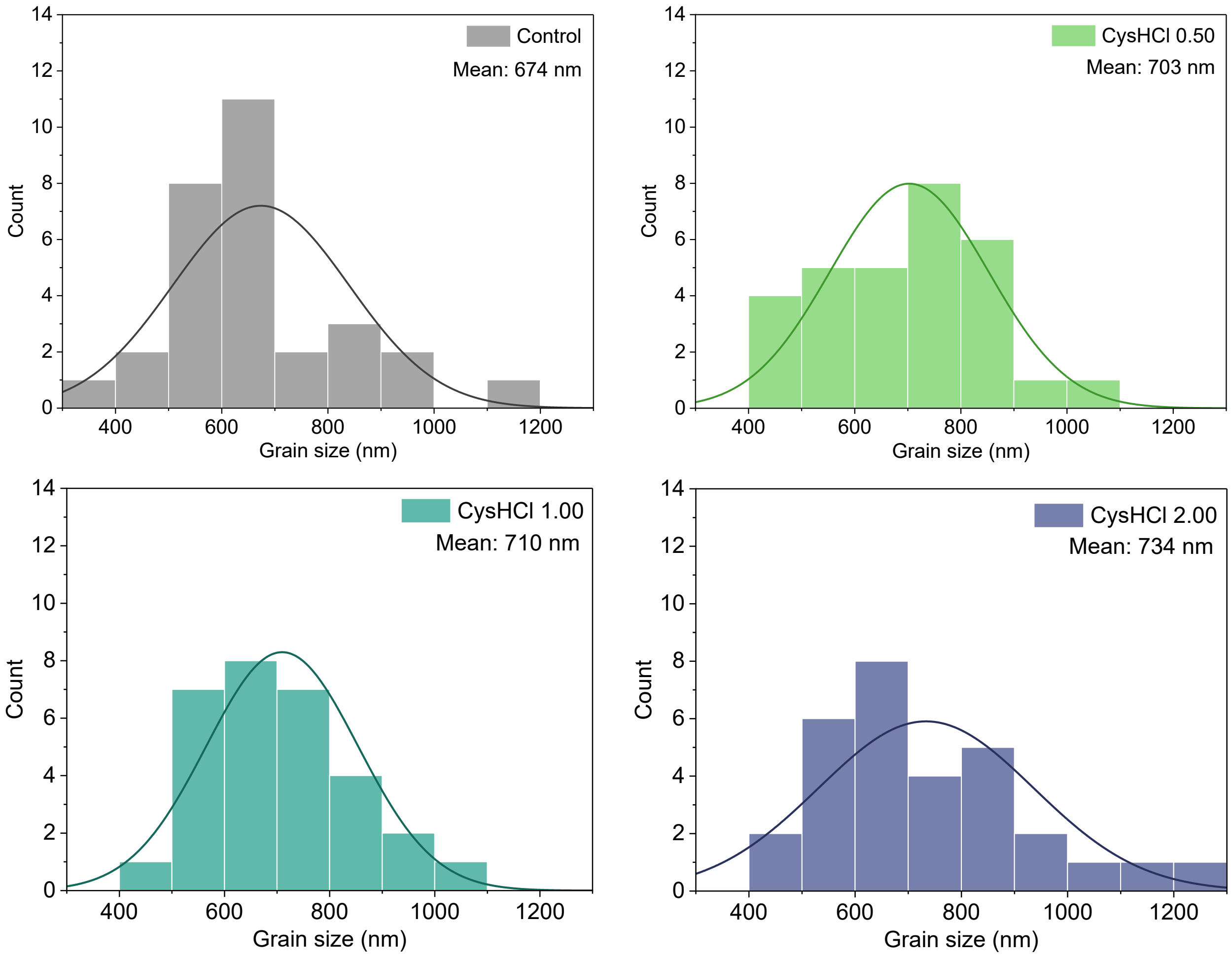

Figure S10. Statistical collection of the apparent grain size of the films prepared with different amounts of CysHCl, based on the SEM images shown in **Figure 3a**.

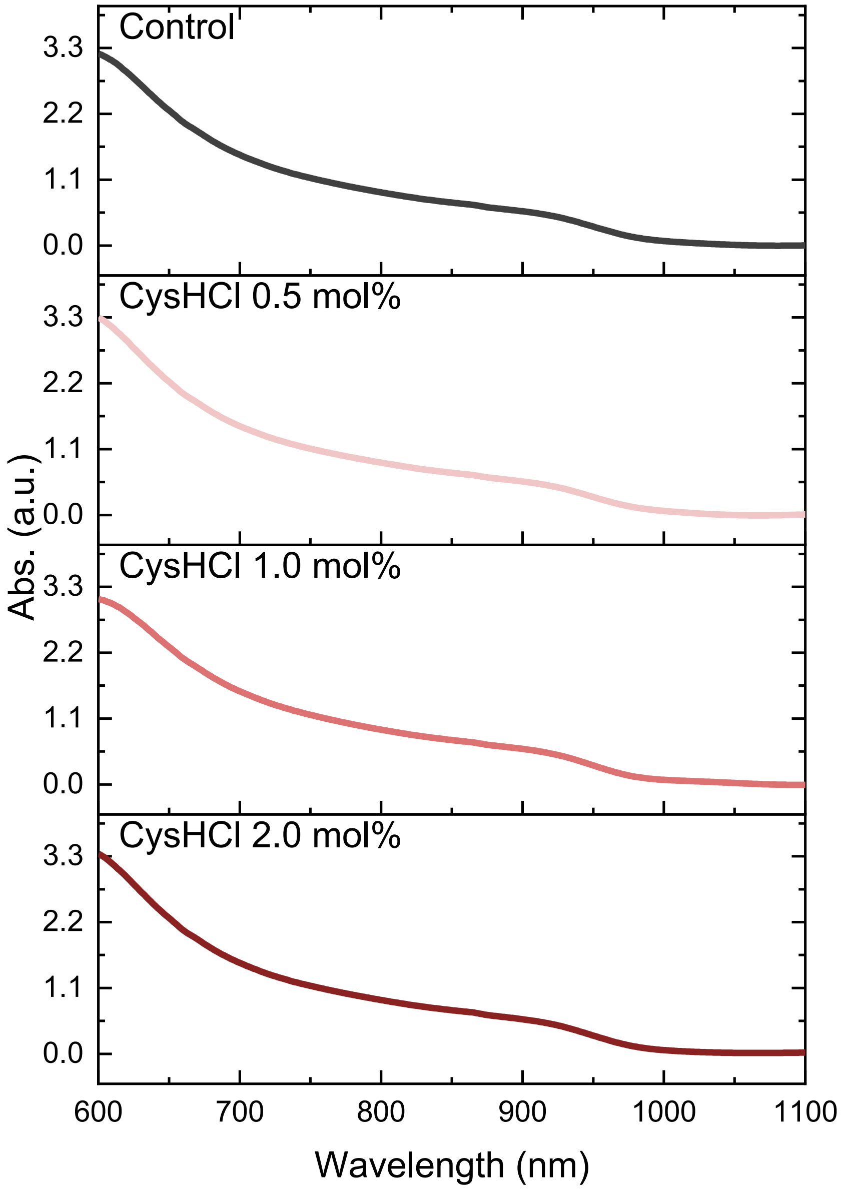

Figure S11. Optical absorption spectra of the perovskite films prepared with different amounts of CysHCl on the quartz substrate.

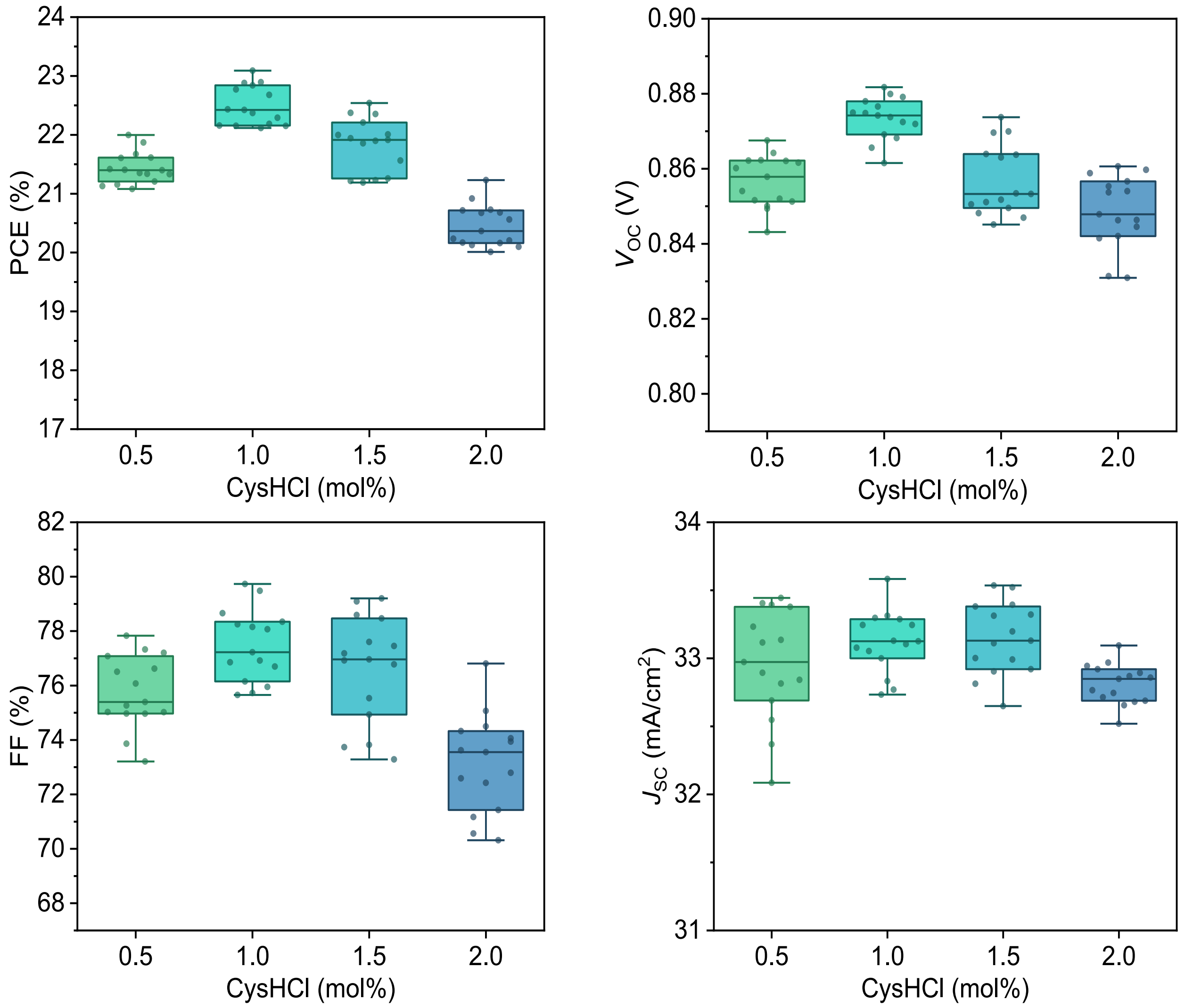

Figure S12. *J–V* parameters of the PSCs with the perovskite films fabricated under different amounts of CysHCl in the same batch, with 15 cells for each condition.

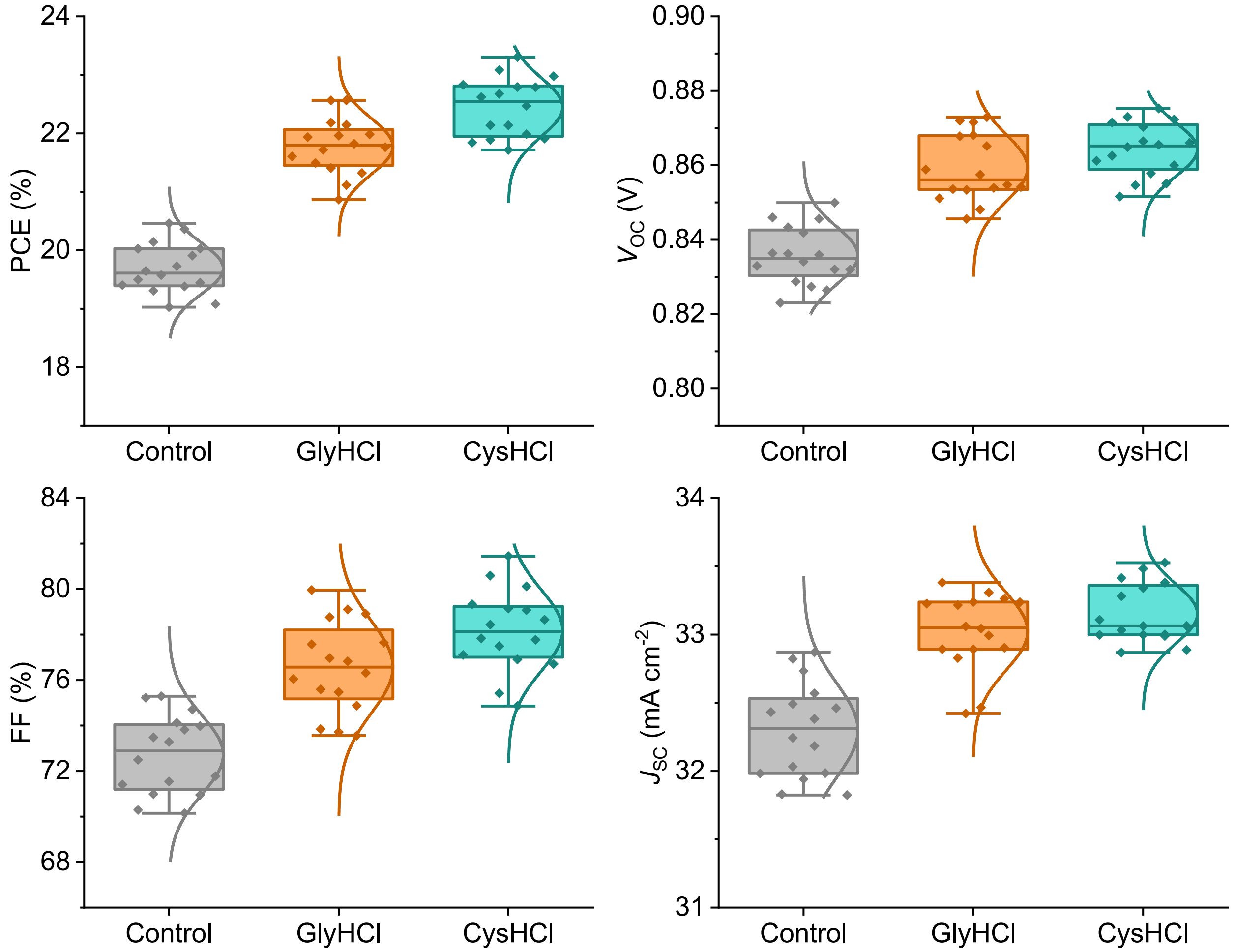

Figure S13. *J–V* parameters of the PSCs with the perovskite films fabricated without a particular additive (Control) and with the addition of 1 mol% GlyHCl and CysHCl in the same batch, with 16 cells for each condition.

References

1 Hu, S. *et al.* Optimized carrier extraction at interfaces for 23.6% efficient tin–lead perovskite solar cells. *Energy Environ. Sci.* **15**, 2096–2107 (2022). https://doi.org/10.1039/d2ee00288d

2 de Mello, J. C., Wittmann, H. F. & Friend, R. H. An improved experimental determination of external photoluminescence quantum efficiency. *Adv. Mater.* **9**, 230–232 (1997). https://doi.org/https://doi.org/10.1002/adma.19970090308

3 Caprioglio, P. *et al.* On the relation between the open-circuit voltage and quasi-Fermi level splitting in efficient perovskite solar cells. *Adv. Energy Mater.* **9**, 1901631 (2019). https://doi.org/https://doi.org/10.1002/aenm.201901631

4 Dasgupta, A. *et al.* Visualizing macroscopic inhomogeneities in perovskite solar cells. *ACS Energy Lett.* **7**, 2311–2322 (2022). https://doi.org/10.1021/acsenergylett.2c01094

5 Krumrey, M. & Ulm, G. High-accuracy detector calibration at the PTB four-crystal monochromator beamline. *Nuclear Instruments and Methods in Physics Research Section A: Accelerators, Spectrometers, Detectors and Associated Equipment* **467-468**, 1175-1178 (2001). https://doi.org/https://doi.org/10.1016/S0168-9002(01)00598-8

6 Hoell, A., Zizak, I., Bieder, H. & Mokrani, L. DE102006029449. (2007).

7 Keiderling, U. The new 'BerSANS-PC' software for reduction and treatment of small angle neutron scattering data. *Appl. Phys. A* **74**, s1455-s1457 (2002). https://doi.org/10.1007/s003390201561

8 Bressler, I., Kohlbrecher, J. & Thunemann, A. F. SASfit: a tool for small-angle scattering data analysis using a library of analytical expressions. *J. Appl. Crystallogr.* **48**, 1587-1598 (2015). https://doi.org/doi:10.1107/S1600576715016544

9 Vrij, A. *et al.* Light scattering of colloidal dispersions in non-polar solvents at finite concentrations. Silic spheres as model particles for hard-sphere interactions. *Faraday Discuss. Chem. Soc.* **76**, 19-35 (1983). https://doi.org/10.1039/DC9837600019

10 Orsi, D. *et al.* Dynamics in dense hard-sphere colloidal suspensions. *Physical Review E* **85**, 011402 (2012). https://doi.org/10.1103/PhysRevE.85.011402

11 Auer, S. & Frenkel, D. Prediction of absolute crystal-nucleation rate in hard-sphere colloids. *Nature* **409**, 1020-1023 (2001). https://doi.org/10.1038/35059035

12 Percus, J. K. & Yevick, G. J. Analysis of Classical Statistical Mechanics by Means of Collective Coordinates. *Phys. Rev.* **110**, 1-13 (1958). https://doi.org/10.1103/PhysRev.110.1

13 Vrij, A. Mixtures of hard spheres in the Percus–Yevick approximation. Light scattering at finite angles. *J. Chem. Phys.* **71**, 3267-3270 (1979). https://doi.org/10.1063/1.438756

14 M. J. Frisch, G. W. T., H. B. Schlegel, G. E. Scuseria, M. A. Robb, J. R. Cheeseman, G. Scalmani, V. Barone, G. A. Petersson, H. Nakatsuji, X. Li, M. Caricato, A. Marenich, J. Bloino, B. G. Janesko, R. Gomperts, B. Mennucci, H. P. Hratchian, J. V. Ortiz, A. F. Izmaylov, J. L. Sonnenberg, D. Williams-Young, F. Ding, F. Lipparini, F. Egidi, J. Goings, B. Peng, A. Petrone, T. Henderson, D. Ranasinghe, V. G. Zakrzewski, J. Gao, N. Rega, G. Zheng, W. Liang, M. Hada, M. Ehara, K. Toyota, R. Fukuda, J. Hasegawa, M. Ishida, T. Nakajima, Y. Honda, O. Kitao, H. Nakai, T. Vreven, K. Throssell, J. A. Montgomery, Jr., J. E. Peralta, F. Ogliaro, M. Bearpark, J. J. Heyd, E. Brothers, K. N. Kudin, V. N. Staroverov, T. Keith, R. Kobayashi, J. Normand, K. Raghavachari, A. Rendell, J. C. Burant, S. S. Iyengar, J. Tomasi, M. Cossi, J. M. Millam, M. Klene, C. Adamo, R. Cammi, J. W. Ochterski, R. L. Martin, K. Morokuma, O. Farkas, J. B. Foresman, and D. J. Fox, Gaussian, Inc.,. Gaussian 09, Revision A.02,. *Gaussian 09, Revision A.02,* (2016).

15 Becke, A. D. Density-functional thermochemistry. III. The role of exact exchange. *J. Chem. Phys.* **98**, 5648-5652 (1993). https://doi.org/10.1063/1.464913

16 VandeVondele, J. *et al.* Quickstep: Fast and accurate density functional calculations using a mixed Gaussian and plane waves approach. *Comput. Phys. Commun.* **167**, 103-128 (2005). https://doi.org/https://doi.org/10.1016/j.cpc.2004.12.014

17 Ernzerhof, M. & Scuseria, G. E. Assessment of the Perdew–Burke–Ernzerhof exchange-correlation functional. *J. Chem. Phys.* **110**, 5029-5036 (1999). https://doi.org/10.1063/1.478401

18 Grimme, S., Ehrlich, S. & Goerigk, L. Effect of the damping function in dispersion corrected density functional theory. *J. Comput. Chem.* **32**, 1456-1465 (2011). https://doi.org/https://doi.org/10.1002/jcc.21759

19 VandeVondele, J. & Hutter, J. Gaussian basis sets for accurate calculations on molecular systems in gas and condensed phases. *J. Chem. Phys.* **127** (2007). https://doi.org/10.1063/1.2770708

20 Stoumpos, C. C., Malliakas, C. D. & Kanatzidis, M. G. Semiconducting Tin and Lead Iodide Perovskites with Organic Cations: Phase Transitions, High Mobilities, and Near-Infrared Photoluminescent Properties. *Inorg. Chem.* **52**, 9019-9038 (2013). https://doi.org/10.1021/ic401215x
